# 5-aminolevulinate and CHIL3/CHI3L1 treatment amid ischemia aids liver metabolism and reduces ischemia-reperfusion injury

**DOI:** 10.7150/thno.83163

**Published:** 2023-08-28

**Authors:** Guanghui Jin, Na Guo, Yasong Liu, Lele Zhang, Liang Chen, Tao Dong, Wei Liu, Xiaomei Zhang, Yong Jiang, Guo Lv, Fei Zhao, Wei Liu, Ziqing Hei, Yang Yang, Jingxing Ou

**Affiliations:** 1 Department of Hepatic Surgery and Liver transplantation Center, the Third Affiliated Hospital of Sun Yat-Sen University; Organ Transplantation Institute, Sun Yat-sen University; Organ Transplantation Research Center of Guangdong Province, Guangdong Province Engineering Laboratory for Transplantation Medicine, Guangzhou, China.; 2 Guangdong Key Laboratory of Liver Disease Research, the Third Affiliated Hospital of Sun Yat-sen University, Guangzhou, China.; 3 Key Laboratory of Liver disease biotherapy and Translational Medicine of Guangdong Higher Education Institutes, the Third Affiliated Hospital of Sun Yat-sen University, Guangzhou, China.; 4 Department of Anesthesiology, the Third Affiliated Hospital of Sun Yat-sen University, Guangzhou, China.; 5 School of Optometry and Ophthalmology and Eye Hospital, Wenzhou Medical University, Wenzhou, Zhejiang, China; The State Key Laboratory of Optometry, Ophthalmology and Vision Science, Wenzhou, Zhejiang, China.

**Keywords:** 5-aminolevulinic acid, CX3CR1, chitinase-like 3, chitinase-3-like protein 1, hepatic ischemia-reperfusion

## Abstract

**Rationale:** Liver resection and transplantation surgeries are accompanied by hepatic ischemia-reperfusion (HIR) injury that hampers the subsequent liver recovery. Given that the liver is the main organ for metabolism and detoxification, ischemia-reperfusion in essence bestows metabolic stress upon the liver and disrupts local metabolic and immune homeostasis. Most of the recent and current research works concerning HIR have been focusing on addressing HIR-induced hepatic injury and inflammation, instead of dealing with the metabolic reprogramming and restoration of redox homeostasis. As our previous work uncovers the importance of 5-aminolevulinate (5-ALA) synthesis during stress adaptation, here we evaluate the effects of supplementing 5-ALA to mitigate HIR injury.

**Methods:** 5-ALA was supplemented into the mice or cultured cells during the ischemic or oxygen-glucose deprivation (OGD) phase. Following reperfusion or reoxygenation, cellular metabolism and energy homeostasis, mitochondrial production of reactive oxygen species (ROS) and transcriptomic changes were evaluated in HIR mouse models or cultured hepatocytes and macrophages. Liver injury, hepatocytic functional tests, and macrophagic M1/M2 polarization were assessed.

**Results:** Dynamic changes in the expression of key enzymes in 5-ALA metabolism were first confirmed in donor and mouse liver samples following HIR. Supplemented 5-ALA modulated mouse hepatic lipid metabolism and reduced ATP production in macrophages following HIR, resulting in elevation of anti-inflammatory M2 polarization. Mechanistically, 5-ALA down-regulates macrophagic chemokine receptor CX3CR1 via the repression of RelA following OGD and reoxygenation (OGD/R). *Cx3cr1* KO mice demonstrated milder liver injuries and more macrophage M2 polarization after HIR. M2 macrophage-secreted chitinase-like protein 3 (CHIL3; CHI3L1 in human) is an important HIR-induced effector downstream of CX3CR1 deficiency. Addition of CHIL3/CHI3L1 alone improved hepatocellular metabolism and reduced OGD/R-inflicted injuries in cultured mouse and human hepatocytes. Combined treatment with 5-ALA and CHIL3 during the ischemic phase facilitated lipid metabolism and ATP production in the mouse liver following HIR.

**Conclusion:** Our results reveal that supplementing 5-ALA promotes macrophagic M2 polarization via downregulation of RelA and CX3CR1 in mice following HIR, while M2 macrophage-produced CHIL3/CHI3L1 also manifests beneficial effects to the recovery of hepatic metabolism. 5-ALA and CHIL3/CHI3L1 together mitigate HIR-induced mitochondrial dysfunction and hepatocellular injuries, which may be developed into safe and effective clinical treatments to attenuate HIR injuries.

## Introduction

Orthotopic liver transplantation is the standard of care for patients with end-stage liver diseases. Liver transplantation, liver resection and other trauma surgeries all require clamping of the liver portal vein, which is then released at the end of the operation. This inevitably leads to hepatic ischemia-reperfusion (HIR) injury that is among the major post-surgical complications affecting patients' quality of life 1. During HIR, drastic changes in oxygen, pH and other physiological conditions disrupt the metabolic homeostasis of the liver, stress the mitochondria and initiate the overproduction of reactive oxygen species (ROS), leading to subsequent hepatic damage and sterile inflammatory response 2, 3. As the majority of research efforts in the field has been dedicated to deciphering the complex processes of HIR-induced cell death and inflammation 2-6, seemingly less attention was paid to attenuate HIR-triggered metabolic stress so as to address the complex issue from the upstream.

Recently, we revealed that the non-proteinogenic amino acid 5-Aminolevulinate (5-ALA) is associated with stress responses to cold storage-rewarming and hypoxia-reoxygenation in the hepatocyte-like cells derived from the induced pluripotent stem cells of a hibernating mammal 7. Addition of 5-ALA into the cold storage solution for human stem cell-derived hepatocyte-like cells could substantially alleviate rewarming-induced mitochondrial ROS overproduction and dysfunction in energy metabolism. It can also repress liver injuries and the expression of inflammation- and allograft rejection-related genes, and improve liver metabolism in extendedly cold-stored rat livers following warm reperfusion. Moreover, we also found in donor split liver samples and mouse liver partial resection and regeneration models that 5-ALA synthesis positively correlates with liver regeneration 8. Interestingly, deficiency of macrophagic heme oxygenase-1 (HO-1/HMOX1), a key enzyme downstream of the 5-ALA metabolic pathway, exacerbates liver inflammation and hepatocellular death in mouse HIR models 9. Pre-treatment with 5-ALA can induce HO-1 and decrease inflammatory response in RAW 264.7 macrophages without affecting their phagocytic activities 10. Thus, we speculated that the availability of 5-ALA during HIR may impact the hepatic metabolism and immune microenvironment, while its molecular underpinnings in the process remain to be elucidated.

Accordingly, we found that 5-ALA supplements modulate the macrophagic expression of several chemokine receptors, including C-X3-C Motif Chemokine Receptor 1 (CX3CR1), and promote hepatic recruitment of circulating monocytes/macrophages following liver partial resection and regeneration 8. Liver resident macrophages, also known as Kupffer cells, are the largest population of the hepatic non-parenchymal cells and crucial for the maintenance of liver immune homeostasis. Upon stressed or injured conditions such as HIR, Kupffer cell population is suppressed, bone marrow-derived monocytes/macrophages rapidly infiltrate 11, 12 and undergo pro-inflammatory (M1) or anti-inflammatory (M2) polarization that requires substantial metabolic reprogramming 13-15. Notably, CX3CR1 is expressed in circulating monocytes, tissue resident macrophages, T cells and dendritic cells 16, known to recruit immune cells 17, 18 and regulate the survival, proliferation and polarization of monocytes and tissue macrophages 19-21.

Thus, we investigated how 5-ALA treatment may impact liver metabolism and macrophage-related inflammatory response following HIR. Here we report that 5-ALA treatment during the ischemic stage alleviated HIR injury and downregulated CX3CR1 in mice. Then, CX3CR1 deficiency upregulated macrophagic secretion of chitinase-like protein 3 (CHIL3). CHIL3 (CHI3L1 in human) proteins are synthesized by various cell types and best known as a biomarker secreted by M2 macrophages in various diseases 22, 23. Surprisingly, we found beneficial effects of CHIL3/CHI3L1 in modulating hepatocellular metabolism and mitochondrial activities following HIR. Hence, a single-dose treatment of 5-ALA and CHIL3/CHI3L1 can significantly improve hepatic metabolic activities and further diminish HIR injury.

## Results

### 5-ALA treatment attenuates HIR injury

**Figure 1:**
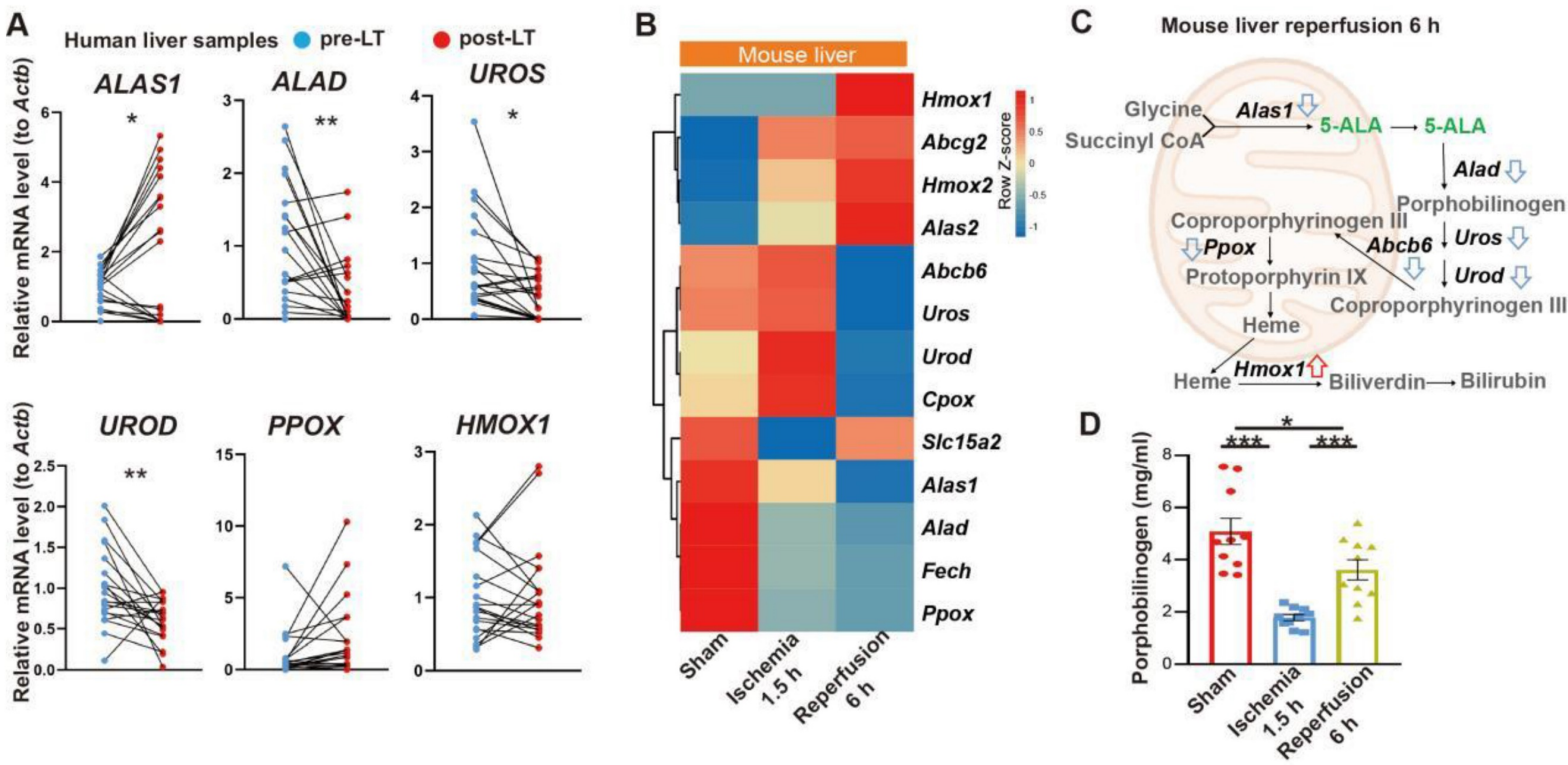
** Liver transplantation and hepatic ischemia-reperfusion (HIR) affect 5-ALA metabolism.** (A) Relative mRNA expression of genes related to the 5-ALA metabolic pathway (*ALAS1, ALAD, UROS, UROD, PPOX, HMOX1*) in human donor liver before (pre-LT) and after (post-LT) transplantation (n = 20). The mRNA level of each gene is normalized to that of* ACTB*. (B) Heatmap of 5-ALA metabolism-related genes expressed at indicated conditions (n = 4 for sham, n = 5 for ischemia 1.5 h and reperfusion 6 h). Red, high relative expression; blue, low relative expression. (C) Diagram summarizing changes in the expression of 5-ALA metabolism-related genes in mice 6 h after HIR. (D) The level of porphobilinogen (PBG; metabolite downstream of 5-ALA) in mouse HIR liver samples at indicated conditions (n = 10). Results are presented as mean ± SEM. *P < 0.05, **P < 0.01, ***P < 0.001 by two-tailed Student's *t* tests (A) or one-way ANOVA (D).

We first looked into possible correlation between 5-ALA metabolism and HIR. The expression of several key enzymes in the 5-ALA metabolic pathway was probed in paired pre- and post-transplantation (LT) samples of human donor livers (Table S1). Interestingly, *5-ALA synthase 1* (*ALAS1*) appeared to be upregulated in the majority of post-LT samples, whilst the downstream enzymes *5-ALA dehydratase* (*ALAD*), *uroporphyrinogen-III synthase* (*UROS*) and *uroporphyrinogen decarboxylase* (*UROD*) were downregulated (Figure 1A). These results imply that elevated 5-ALA synthesis may be needed right after LT. Revealed by transcriptomic analysis, mouse HIR operation (Figure S1A) also led to complex changes in the expression of genes related to 5-ALA metabolism (Figures 1B-C and Data S1). *Alas1* and *Alad* expression in mouse liver was repressed at both transcript (Figure 1B) and protein (Figure S1B) levels 6 h after HIR, whilst *Hmox1* transcription was elevated (Figure 1B and Figure S1C). Because we have not found a quantitative biochemical method for 5-ALA, instead, the liver content of porphobilinogen (PBG), the metabolite immediately downstream of 5-ALA metabolism (Figure 1C), was measured in mice. Consistent with IR-associated changes in ALAD protein levels and enzymatic activities (Figure S1B and D), the hepatic PBG level declined during ischemia and then partially recovered shortly after reperfusion (Figure 1D). Taken together, the upstream suppression of 5-ALA metabolic activities and downstream elevation of *Hmox1* expression in mice following HIR may result in deficiency of important metabolites in the 5-ALA metabolic pathway, hence contributing to metabolic dysfunction or stress in the mouse liver.

**Figure 2:**
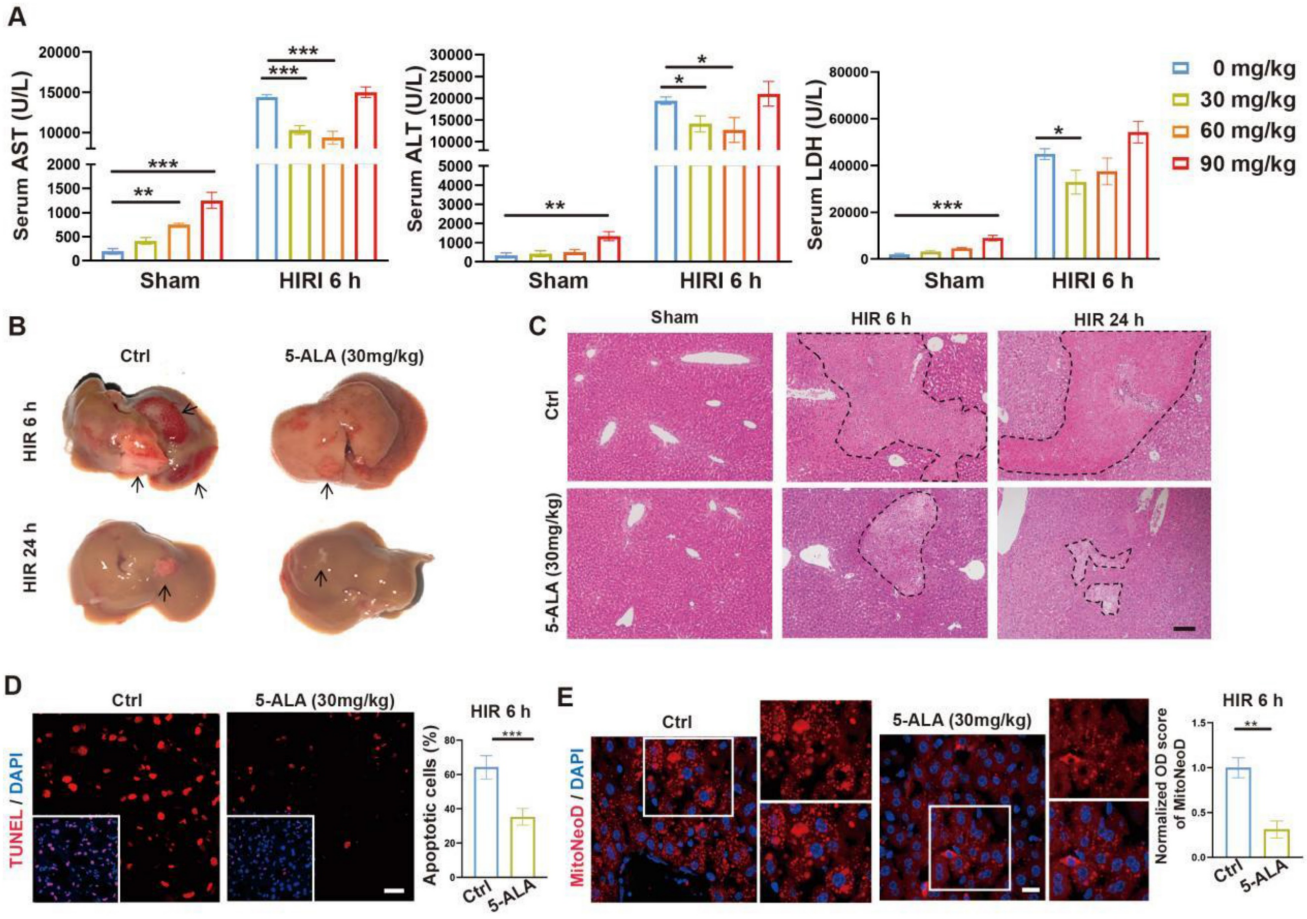
** 5-ALA treatment at the ischemic phase alleviates HIR-induced injury and mitochondrial ROS overproduction.** (A) Liver injury indicators AST, ALT and LDH levels in mice at indicated conditions (n = 6 per group). (B) Gross appearance of mouse livers at indicated conditions (arrows denote areas of HIR injury). (C) Representative H&E staining of mouse liver sections at indicated conditions (areas of HIR injury are marked by broken lines; scale bar = 0.1 mm). (D) TUNEL (red) and DAPI (blue) staining and quantitative analysis of apoptotic cells in mouse liver sections at indicated conditions (n = 5, scale bar = 10 μm). (E) MitoNeoD (red) and DAPI (blue) staining to evaluate mitochondrial ROS overproduction in mouse liver sections at indicated conditions (n = 5, scale bar = 10 μm). Results are presented as mean ± SEM. *P < 0.05, **P < 0.01, ***P < 0.001 by two-tailed Student's *t* tests (D) and (E) or one-way ANOVA (A).

To test our hypothesis, we then performed intraperitoneal (i.p.) injection of 5-ALA in mice during the ischemia phase, and observed the outcome 6 or 24 h after reperfusion (Figure S2A). A single dose of 30 mg/kg 5-ALA demonstrated liver protective effects and was used throughout this study (Figure 2A-C and S2B-C). As expected 7, 5-ALA supplement attenuated HIR-induced cell apoptosis (Figure 2D) and mitochondrial ROS overproduction visualized by MitoNeoD staining 24 in the mouse liver (Figure 2E). Judging by the morphology of the cells positively stained with MitoNeoD, we then performed 4 h of oxygen-glucose deprivation followed by 6 h of reoxygenation (OGD/R) on mouse primary hepatocyte and hepatic macrophage cultures, confirming that such HIR-like stress triggers mitochondrial ROS overproduction in these cell types and can be alleviated by 5-ALA (Figure S2D).

### 5-ALA treatment modulates hepatic metabolism and macrophagic M2 polarization following HIR

**Figure 3:**
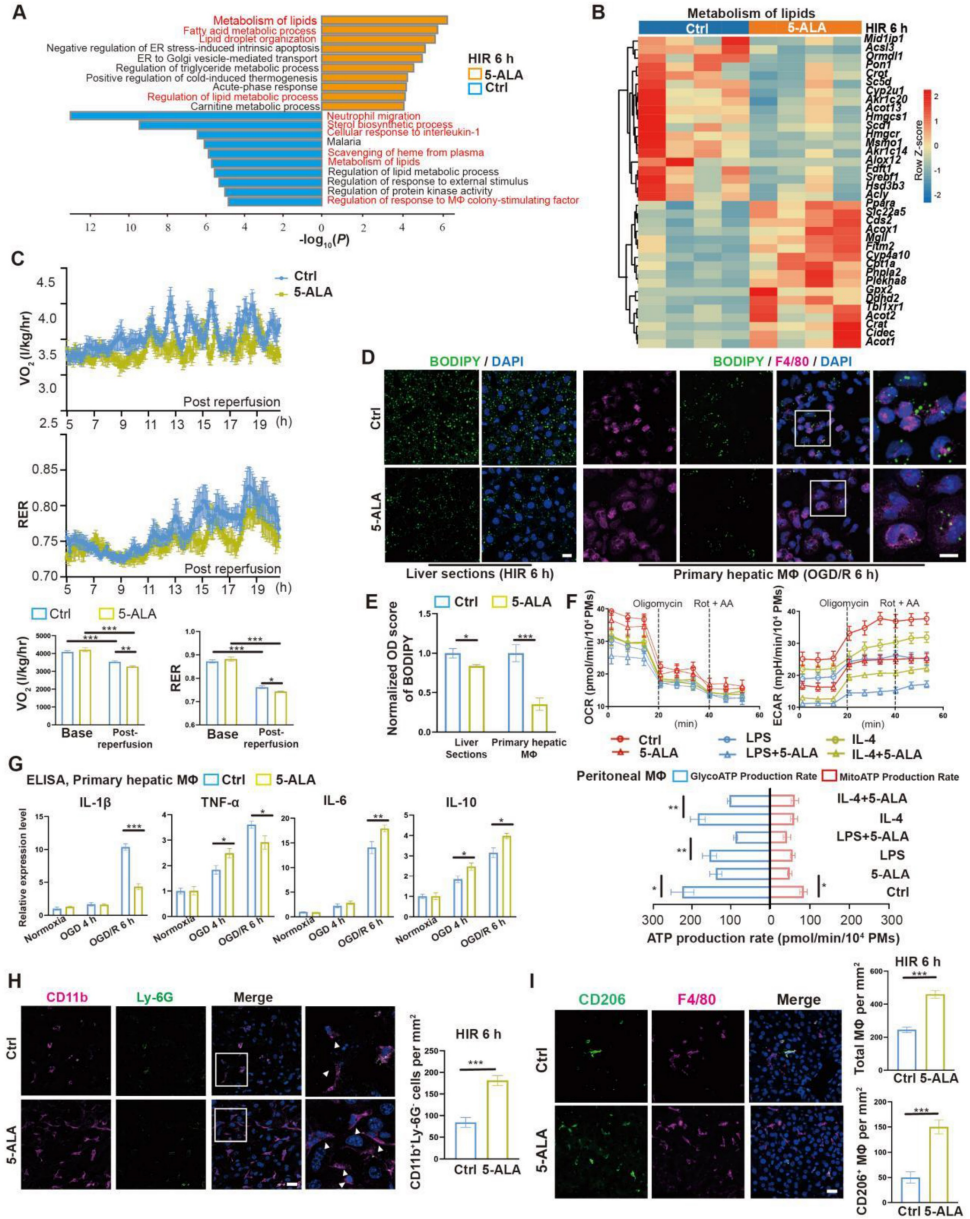
** 5-ALA treatment facilitates hepatic lipid metabolism and macrophagic M2 polarization and infiltration.** (A) Enrichment analysis on differentially expressed genes (DEGs) in mouse liver samples 6 h after HIR. Orange, upregulated in 5-ALA-treated mice; blue, downregulated in 5-ALA-treated mice. (B) Heatmap of DEGs related to metabolism of lipids (R-MMU-556833) 6 h after HIR (n = 4 per group). Red, high relative expression; blue, low relative expression. Also see Data S2. (C) Up: Oxygen consumption rates (OCR, VO_2_) and respiratory exchange ratio (RER) at annotated conditions; down: average OCR (VO_2_) and RER at annotated conditions. Values are normalized to the body weight of the mice (n = 6 per group). (D) BODIPY (green), mouse macrophagic marker F4/80 (magenta) and DAPI (blue) staining of mouse liver sections or cultured mouse hepatic macrophages (MΦ) showing the dynamic changes of lipid droplets at indicated conditions. OGD/R, oxygen-glucose deprivation and reoxygenation (Scale bar = 10 μm). (E) Quantification of BODIPY fluorescence intensity from (D) (n = 5 for mouse liver sections; n = 8 for hepatic macrophage cultures). (F) Up: OCR and extracellular acidification rates (ECAR) measured in mouse peritoneal macrophage cultures in control (ctrl), LPS-induced M1 polarization, or IL-4-induced M2 polarization group; down: calculated ATP production rates at indicated conditions (n = 8 per group). (G) ELISA of IL-1β, IL-6, IL-10 and TNF-α in medium supernatant of primary hepatic macrophage cultures at indicated conditions (n = 6). (H) Left: Immunofluorescent staining of DAPI (blue), CD11b (magenta) and Ly-6G (green) in mouse liver sections at indicated conditions (white arrows denote CD11b^+^ Ly-6G^-^ cells, scale bar = 10 μm); right: density of CD11b^+^ Ly-6G^-^ cells in these liver sections (n = 8). (I) Left: Immunofluorescent staining of DAPI (blue), F4/80 (magenta) and M2 macrophagic marker CD206 (green) in mouse liver sections at indicated conditions (scale bar = 10 μm); right: density of total macrophages (F4/80^+^) and M2 macrophages (F4/80^+^ CD206^+^) in these liver sections (n = 5). Results are presented as mean ± SEM. *P < 0.05, **P < 0.01, ***P < 0.001 by two-tailed Student's *t* tests (E), (H) and (I) or one-way ANOVA (C) and (G).

Prompted by the above results, enrichment analysis on the effects of 5-ALA on the mouse liver transcriptome after HIR was performed (Figure 3A and Data S2). Among the top enriched pathways, genes related to lipid metabolic activities were profoundly and differentially regulated by the supplemented 5-ALA (Figure 3B). Among the downregulated pathways, 5-ALA treatment appeared to affect scavenging of heme from plasma, and suppress neutrophil migration and responses to interleukin-1 (IL-1) and macrophage colony-stimulating factor (Figure S3A-C). The notion that 5-ALA may mediate hepatic immune responses during HIR is consistent with our recent findings that 5-ALA affects the expression of genes related to immune responses in rat livers following cold storage 7 (Figure S3D), and mediates the expression of several chemokine receptors and macrophagic anti-inflammatory (M2-like) polarization in mouse liver partial resection and regeneration models 8.

Next, the effects of 5-ALA on mouse metabolism during HIR were monitored with the metabolic cage system (Figure S3E). Mouse oxygen consumption (VO_2_) and respiratory exchange ratio (RER) appeared to decline following HIR (Figure S3F), and were further downregulated by the 5-ALA treatment (Figure 3C). These results suggested that mouse energy expenditure is suppressed by HIR, and 5-ALA promotes fat burning in the post-reperfusion phase. This was further supported by BODIPY staining, which revealed diminished accumulation of lipid droplets in liver sections from 5-ALA-treated mice after HIR, and in mouse primary hepatic macrophage cultures 6 h after OGD/R (Figure 3D-E).

We next evaluated whether 5-ALA affects macrophagic energy metabolism and polarization. ATP production from mitochondrial oxidative phosphorylation or from glycolysis was measured in cultured mouse primary peritoneal macrophages (Figure 3F). Intriguingly, 5-ALA suppressed ATP production from both pathways in M0 cultures, and also reduced glycolic ATP production in lipopolysaccharide (LPS)-induced M1 and interleukin-4 (IL-4)-induced M2 polarization. In contrast, 5-ALA showed little effect on mitochondrial ATP production during macrophagic polarization. Subsequently, quantitative PCR (qPCR) assays suggested that OGD/R stimuli led to significantly higher expression of *Il1β*, *Inos1*, *Tnfa*, *Il6* and *Il10* in hepatic macrophages than that in hepatocytes (Figure S3G). 5-ALA promoted a higher expression of M2 signature genes *Cd206*, *Il10* and *Arg1*, suppressed the elevation of M1-related *Inos1* and *Il1β* gene expression, whilst upregulated *Il6* expression in macrophages following OGD/R (Figure S3H). Similar results were observed by ELISA of IL-1β, IL-6 and IL-10 in OGD/R-stressed hepatic macrophages (Figure 3G). *In vivo*, more CD11b^+^Ly-6G^-^ myeloid-derived cells had been recruited to the liver 6 h after HIR in 5-ALA-treated mice (Figure 3H). Meanwhile, 5-ALA treatment also resulted in higher density of macrophages in the liver, among which more F4/80^+^CD206^+^ M2 macrophages were identified compared to that in the control group (Figure 3I). Taken together, 5-ALA treatment may attenuate HIR injury in mice via modulation of hepatic metabolism and macrophagic ATP production, promoting an anti-inflammatory microenvironment for recovery.

### CX3CR1 functions downstream of 5-ALA treatment in HIR

**Figure 4:**
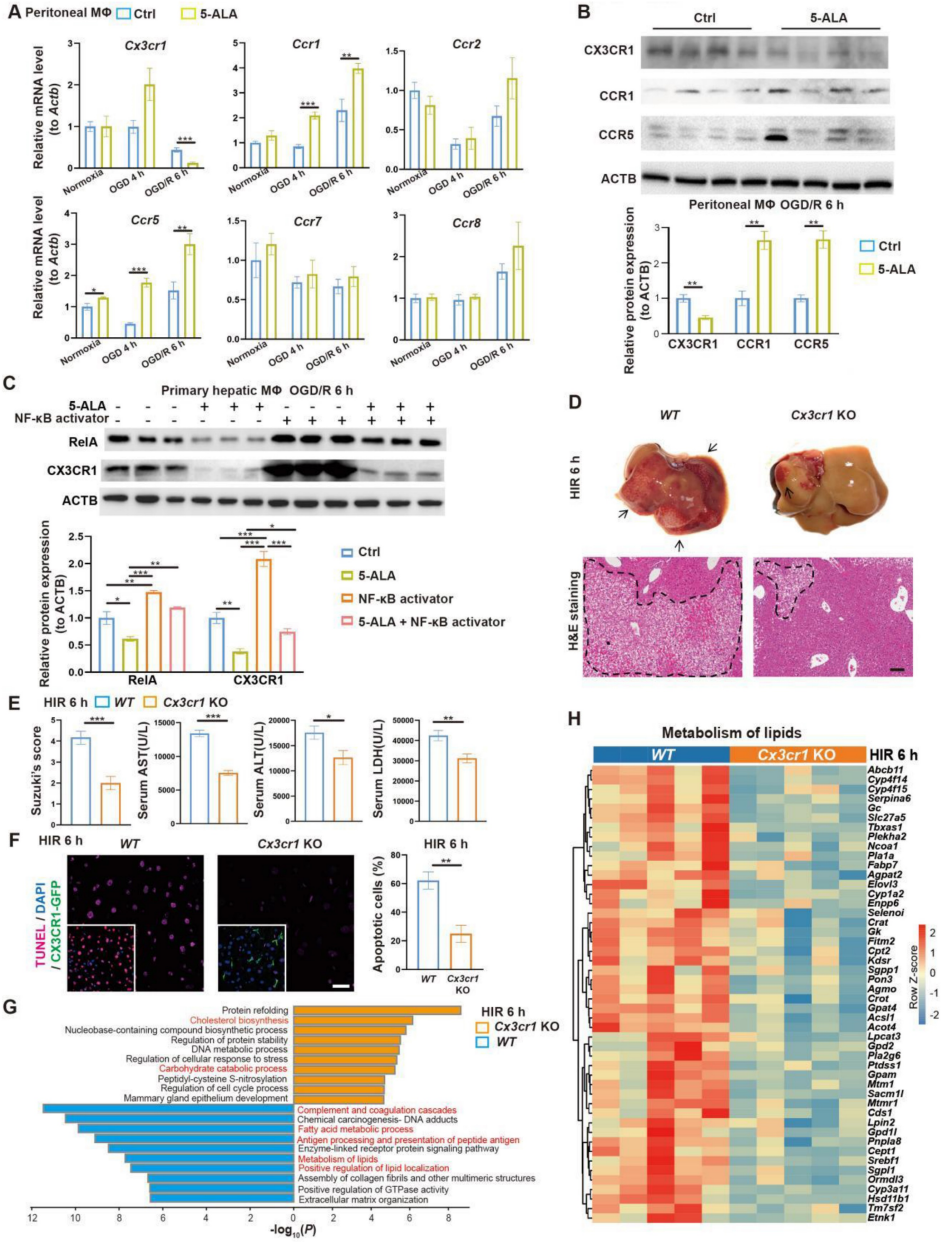
** 5-ALA inhibits the RelA-CX3CR1 pathway to reprogram hepatic metabolism and attenuate HIR injury in mice.** (A) Relative mRNA expression of chemokine receptors (*Cx3cr1, Ccr1, Ccr2, Ccr5, Ccr7, Ccr8*) in mouse macrophage cultures at indicated conditions (n = 6). The mRNA level of each gene is normalized to that of* Actb*. (B) Representative western blots of chemokine receptor proteins (CX3CR1, CCR1, CCR5) in mouse macrophage cultures 6 h after OGD/R (n = 3). Their signal intensity is normalized to that of ACTB. (C) Representative western blots of RelA (p65 NF-κB) and CX3CR1 in mouse primary hepatic macrophage cultures at indicated conditions (NF-κB activator: 8 μg/mL betulinic acid, 5-ALA: 1 mM, n = 3). Their signal intensity is normalized to that of ACTB. (D) Up: gross appearance of mouse livers following HIR (arrows denote areas of HIR injury); down: H&E staining of liver sections (areas of HIR injury are labeled by broken lines; scale bar = 0.1 mm) from *WT* and *Cx3cr1* KO mice at indicated conditions. (E) Suzuki's score, AST, ALT and LDH levels in *WT* and *Cx3cr1* KO mice 6 h after HIR (n = 6 for Suzuki's score; n = 12 for AST, ALT and LDH measurements). (F) CX3CR1-GFP (green), TUNEL (red), and DAPI (blue) fluorescence in mouse liver sections at indicated conditions (n = 5; scale bar = 10 μm). (G) Enrichment analysis on DEGs in* WT* and *Cx3cr1* KO mouse liver samples following HIR. Orange, upregulated in *Cx3cr1* KO mice; blue, downregulated in *Cx3cr1* KO mice. (H) Heatmap of DEGs related to metabolism of lipids (R-MMU-556833) at indicated conditions (n = 5 per group). Red, high relative expression; blue, low relative expression. Results are presented as mean ± SEM. *P < 0.05, **P < 0.01, ***P < 0.001 by two-tailed Student's *t* tests (B), (E) and (F) or one-way ANOVA (A) and (C).

To search for downstream effectors of 5-ALA on hepatic metabolism and immune milieu in mice subjected to HIR, we first found that 5-ALA treatment augmented chemokine receptors CCR1 and CCR5, whilst downregulated CX3CR1 at the transcript and protein levels after HIR or OGD/R (Figure 4A-B). Although CX3CR1 functions have been well studied in many other disease models, known to recruit circulating leukocytes and mediate M1/M2 macrophage polarization, its action on M0 to M1 or M2 transition is context-dependent 25-28. Here we focused on CX3CR1, as its role in mouse HIR models has yet to be recognized.

We first investigated how 5-ALA supplement may mediate CX3CR1 functions in HIR or OGD/R experiments. RelA (p65 NF-κB) activation has been reported to upregulate CX3CR1 expression 29, 30. In mouse hepatic macrophage cultures, after 6 h of OGD/R, 5-ALA supplement suppressed RelA expression at transcript and protein levels, and antagonized the effects of NF-κB agonists on the expression of *Cx3cr1* and M1 macrophage-enriched *Il1β* (Figure 4C and S4A). Intriguingly, 5-ALA also attenuated the NF-κB agonist-repressed expression of M2 marker *Arg1* (Figure S4A). These results imply that 5-ALA effectors other than RelA also exist and contribute to macrophage polarization. In contrast, the TGF-β1 pathway has also been suggested to enhance CX3CR1 functions 31, but 5-ALA treatment showed no repression on mouse hepatic TGF-β1 levels following HIR (Figure S4B). Taken together, CX3CR1 is likely downregulated through the suppression of RelA activities by 5-ALA during HIR or OGD/R.

To verify the role of CX3CR1 in HIR, HIR surgery was operated on *wild-type* (*WT*) and *Cx3cr1* knockout (*Cx3cr1* KO) mice, subsequent phenotypes evaluated (Figure S4C). Remarkably, *Cx3cr1* KO mice manifested milder HIR injury and hepatic cell apoptosis compared to *WT* (Figure 4D-F). Transcriptomic analysis suggested that CX3CR1 deficiency resulted in the repression of complement cascades, antigen processing and fatty acid/lipid metabolic activities, and facilitated carbohydrate catabolism and cholesterol biosynthesis (Figure 4G-H, Figure S4D-G and Data S1). Hence in mouse HIR models, CX3CR1 not only functions as a modulator for sterile inflammatory response, but surprisingly may also affect the hepatic metabolic activities during recovery.

**Figure 5:**
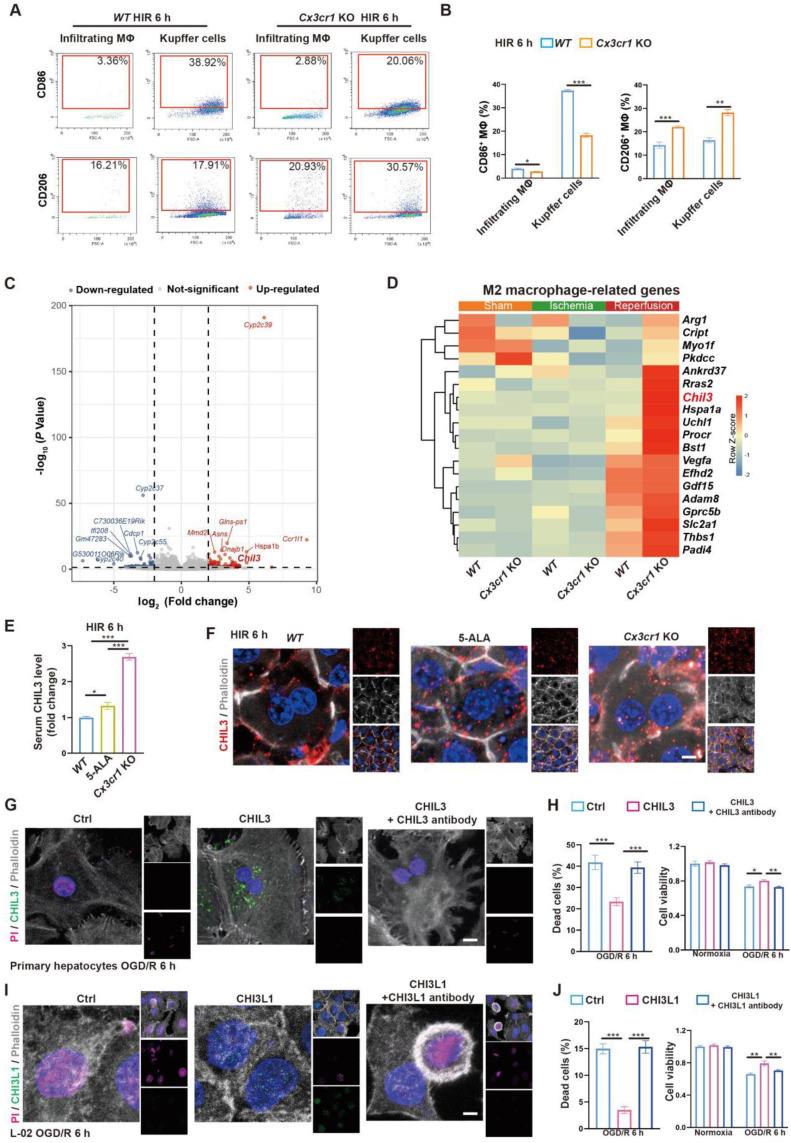
** M2 macrophage-produced CHIL3/CHI3L1 supports hepatocyte survival from OGD/R or HIR.** (A) Flow cytometry analysis of M1 (CD45^+^F4/80^+^CD86^+^) and M2 (CD45^+^F4/80^+^CD206^+^) macrophages in* WT* and *Cx3cr1* KO mouse livers at indicated conditions. (B) Percentage of M1 (CD45^+^F4/80^+^CD86^+^) and M2 (CD45^+^F4/80^+^CD206^+^) macrophages in* WT* and *Cx3cr1* KO mouse livers at indicated conditions (n = 6). (C) Volcano plot of DEGs in *WT* versus *Cx3cr1* KO mouse livers 6 h after HIR (red, upregulated genes in *Cx3cr1* KO; blue, downregulated genes in *Cx3cr1* KO; n = 5). (D) Expression heatmap of M2 macrophage-enriched genes in *WT* and *Cx3cr1* KO mouse livers 6 h after HIR (n = 5). Red, high relative expression; blue, low relative expression. (E) ELISA of mouse serum CHIL3 levels at indicated conditions (n = 6). (F) Representative confocal images of DAPI (blue), CHIL3 (red) and phalloidin (gray) fluorescence in mouse liver sections at indicated conditions (scale bar = 10 μm). (G) Representative confocal images of DAPI (blue), CHIL3 (green), propidium iodide (PI; magenta; staining the nucleus of dead cells) and phalloidin (gray) fluorescence in cultured mouse primary hepatocytes at indicated conditions (scale bar = 10 μm). (H) PI-positive (left) cells from (E) and cell viability tests (right) of mouse primary hepatocytes at indicated conditions (n = 8). (I) Representative confocal images of DAPI (blue), CHIL3 (green), PI (magenta) and phalloidin (gray) fluorescence in cultured human hepatocyte line L-02 at indicated conditions (scale bar = 10 μm). (J) PI-positive cells from (G) and cell viability tests (right) of L-02 cells at indicated conditions (n = 8). Results are presented as mean ± SEM. *P < 0.05, **P < 0.01, ***P < 0.001 by one-way ANOVA (B), (E), (H) and (J).

Next, we looked into CX3CR1 functions in hepatic macrophage polarization in mouse HIR. Compared to that in *WT*, CX3CR1 deficiency led to less M1-like (F4/80^+^CD86^high^) and more M2-like (F4/80^+^CD206^high^) polarization 6 h after HIR in both resident (CD11b^low^Ly-6C^-^) and infiltrating (CD11b^+^Ly-6C^+^) macrophages (Figure 5A-B and Figure S5A-B). Transcriptomic analysis further revealed that the expression of genes enriched in M1 macrophages 32 was downregulated, whilst that enriched in M2 macrophages 32 was further augmented in *Cx3cr1* KO livers 6 h after HIR (Figure 5C-D, Figure S5C and Data S1).

Taken together, in mouse HIR 5-ALA facilitates macrophagic anti-inflammatory M2 polarization likely through the repression of RelA and hence CX3CR1 functions. Notably however, CX3CR1 deficiency appears to also profoundly impact various hepatic metabolic activities. The responsible effectors of 5-ALA/CX3CR1 deficiency in reprogramming liver metabolism were yet to be determined.

### CX3CR1 deficiency enhances CHIL3 to modulate hepatocellular metabolism in HIR

From the transcriptomic analysis, we noted that the post-HIR expression of *Chil3*, also known as *Ym1*, was upregulated in the liver of *Cx3cr1* KO mice (Figure 5C-D and Data S1). CHIL3 is a lectin secreted by macrophages and considered a M2-specific marker. It was also found at elevated levels and associated with the protective immune milieu in the brain of *Cx3cr1* KO mice following brain ischemia-reperfusion 25. Hence, we sought to investigate CHIL3 functions in HIR.

Firstly, serum ELISA revealed the presence of elevated CHIL3 in 5-ALA-treated and *Cx3cr1* KO mice 6 h after HIR (Figure 5E). Western blotting also confirmed higher levels of CHIL3 in* Cx3cr1* KO mouse liver samples 6 h after HIR (Figure S5D). *Chil3* expression was found upregulated in the liver of 5-ALA treated mice during ischemia and 2 h after HIR (Figure S5E), and in cultured mouse hepatic macrophages supplemented with 1-20 mM 5-ALA (Figure S5F). Thus, CHIL3 may serve as a downstream effector of 5-ALA treatment. Then, confocal imaging of CHIL3 immunofluorescent staining on post-HIR mouse liver sections uncovered numerous CHIL3 puncta seemingly internalized by the hepatocytes in 5-ALA-treated or *Cx3cr1* KO mice 6 h after HIR (Figure 5F). In contrast, such distribution of CHIL3 proteins diminished 24 h after HIR (Figure S5G). Next, OGD/R experiments were performed in *WT* mouse primary hepatocyte cultures supplemented with CHIL3 or a premix of CHIL3 and an antibody against CHIL3. Revealed by propidium iodide (PI; staining the nucleus of dead cells) and cell viability assays in cultures stressed by OGD/R, supplemented CHIL3 could be transported into the mouse hepatocytes and enhance cell survival, whilst such effects of CHIL3 were neutralized by the CHIL3 antibody (Figure 5G-H).

**Figure 6:**
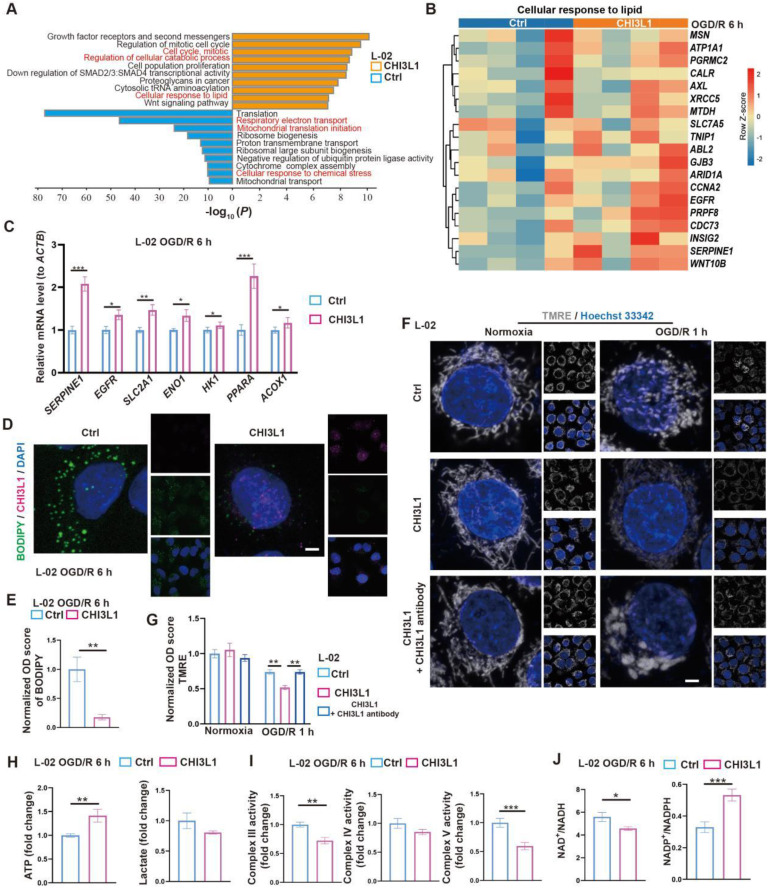
** CHIL3/CHI3L1 profoundly modulates hepatocytic metabolism following OGD/R.** (A) Enrichment analysis on DEGs in control (ctrl) and CHI3L1-treated L-02 cells 6 h after OGD/R. Orange, upregulated in CHI3L1-treated L-02 cells; blue, downregulated in CHI3L1-treated L-02 cells. (B) Heatmap of DEGs related to cellular response to lipid (GO:0071396) at indicated conditions (n = 4 per group). Red, high relative expression; blue, low relative expression. (C) Relative mRNA expression of selected genes related to metabolism (*SERPINE1, EGFR, SLC2A1, ENO1, HK1, PPARA, ACOX1*) in L-02 cells 6 h after OGD/R (n = 12). The mRNA level of each gene is normalized to that of* ACTB*. (D) Representative confocal images of BODIPY (green), CHI3L1 (magenta) and DAPI (blue) staining in L-02 cells at annotated conditions (scale bar = 10 μm). (E) Quantification of BODIPY fluorescence intensity from (D) (n = 5). (F) Representative confocal images of TMRE (gray; fluorescent intensity correlates with mitochondrial membrane potential) and Hoechst 33342 (blue) staining of live L-02 cells at annotated conditions (scale bar = 10 μm). (G) Quantification of TMRE fluorescence intensity from (F) (n = 7). (H) Cellular total ATP level (n = 9) and lactate level (n = 9) in L-02 cultures at annotated conditions. (I) Activities of mitochondrial complex III, IV and V measured in L-02 cells at annotated conditions (n = 9 per group). (J) The ratios of NAD^+^/NADH and NADP^+^/NADPH measured in L-02 cells at annotated conditions (n = 9 per group). Results are presented as mean ± SEM. *P < 0.05, **P < 0.01, ***P < 0.001 by two-tailed Student's *t* tests (C), (E), (H), (I) and (J) or one-way ANOVA (G).

To test the relevance of our findings in humans, we first confirmed that CHI3L1, the human homolog for CHIL3 (Figure S6A), was presented at elevated levels in the serum samples from post-LT patients (Figure S6B and Table S1). Then, similar pro-survival and growth effects of CHI3L1 were verified in OGD/R experiments with human hepatocyte line L-02 (Figure 5I-J and Figure S6C). Transcriptomic and qPCR analyses on cells from these experiments suggested that among the enriched cellular pathways, CHI3L1 promoted cellular catabolism and lipid metabolism, whilst downregulated ribosome biogenesis, mitochondrial respiratory electron transport and proton transport in L-02 (Figure 6A-C, Figure S6D-G and Data S3). Indeed, BODIPY and CHI3L1 staining showed that lipid droplets diminished in L-02 cells with internalized CHI3L1 6 h after OGD/R (Figure 6D-E), implying higher levels of lipid catabolism in these CHI3L1-treated cells. Consistent with the transcriptomic analysis, TMRE staining indicated that mitochondria in CHI3L1-treated cells were more depolarized 1 h after OGD/R, whilst mitochondria in cells of the control group or treated with CHI3L1/antibody against CHI3L1 demonstrated a more spherical shape, differing from that prior to OGD/R stress (Figure 6F-G) and suggestive of abnormal mitochondrial morphology. Next, the effects of CHI3L1 on L-02 cellular energy metabolism and redox homeostasis following OGD/R were investigated. In CHI3L1-treated cells, total ATP production was increased, lactate production was not significantly impacted, while mitochondrial Complex III and V activities were decreased (Figure 6H-I). As indicators of cellular redox homeostasis and energy metabolism 33, the ratio of NAD^+^/NADH decreased, while that of NADP^+^/NADPH increased in CHI3L1-treated L-02 cells 6 h after OGD/R (Figure 6J and Figure S6H).

Overall, our data indicate that CHIL3/CHI3L1 supports mouse and human hepatocyte survival from OGD/R probably through unexpected and complex involvement in hepatocellular metabolic activities. Hepatocytes taking up CHIL3/CHI3L1 proteins probably recover better from OGD/R as the cellular energy demands can be met with enhanced ATP production without stressing out the mitochondrial oxidative phosphorylation pathway.

### Combined treatment of 5-ALA and CHIL3 further mitigates HIR injury

**Figure 7:**
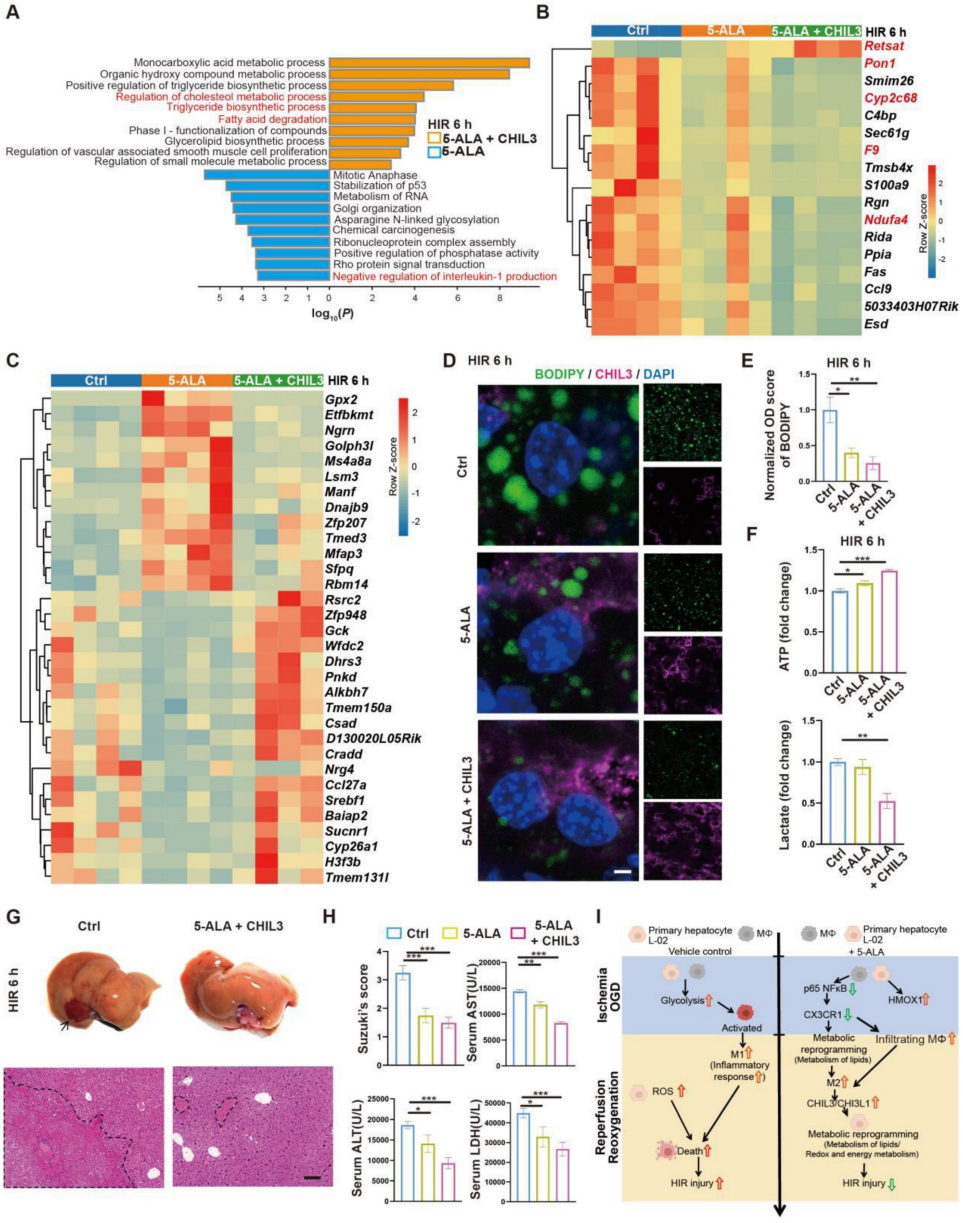
** Combined treatment of 5-ALA and CHIL3 further mitigates HIR injury.** (A) Enrichment analysis on DEGs in mouse liver samples following HIR and indicated treatments. Orange, upregulated in 5-ALA + CHIL3 group; blue, downregulated in 5-ALA + CHIL3 group. (B) Heatmap of DEGs showing similar trends between the 5-ALA treatment group and the 5-ALA + CHIL3 group versus control (ctrl) (n = 4 per group). Red, high relative expression; blue, low relative expression. Also see Figure S5I and Data S2. (C) Heatmap of DEGs showing opposite trends between the 5-ALA treatment group and the 5-ALA + CHIL3 group versus control (ctrl) (n = 4 per group). Red, high relative expression; blue, low relative expression. Also see Figure S5I and Data S2. (D) Representative confocal images of BODIPY (green), CHIL3 (magenta) and DAPI (blue) fluorescence in mouse liver sections at indicated conditions (scale bar = 10 μm). (E) Quantification of BODIPY fluorescence intensity from (D) (n = 5). (F) Total cellular ATP level (n = 9) and lactate level (n = 9) in mouse liver samples from annotated conditions. (G) Up: gross appearance of mouse livers following HIR (arrow denotes area of HIR injury); down: H&E staining of liver sections (areas of HIR injury are labeled by broken lines; scale bar = 0.1 mm) from mice at indicated conditions. (H) Suzuki's score, AST, ALT and LDH levels in mice with indicated conditions 6 h after HIR (n = 8 per group). (I) Schema showing the protective mechanism of 5-ALA and CHIL3/CHI3L1 in HIR injury. ROS, Reactive oxygen species; OXPHOS, mitochondrial oxidative phosphorylation; HIR, hepatic ischemia/reperfusion; OGD, oxygen-glucose deprivation. Results are presented as mean ± SEM. *P < 0.05, **P < 0.01, ***P < 0.001 by one-way ANOVA (E), (F) and (H).

As 5-ALA supplement appeared to enhance *Chil3* expression at high concentrations and within a few hours in OGD/R models (Figure S5E-F), a practical strategy to treat HIR without the need to increase the dosage of 5-ALA or rely on CX3CR1-antagonizing reagents could be a combined treatment of 5-ALA and CHIL3 (Figure S7A). Firstly, we found that in mouse HIR models, CHIL3 injection alone did not have notable effect on attenuating liver injury (Figure S7B-D), probably because the dose of CHIL3 we used was insufficient to overcome the HIR-induced pro-inflammatory microenvironment. Next, transcriptomic analysis indicated that compared to 5-ALA treatment alone, CHIL3 demonstrated its synergetic effects in enhancing hepatic metabolism of fatty acids/lipids (Figure 7A and Figure S7E-F). Compared to the control group, genes showing similar or opposite trends of differential regulation between the 5-ALA and 5-ALA/CHIL3 group were also noted (Figure 7B-C and S7G). As expected, the hepatocellular content of lipid droplets significantly diminished, ATP level increased, and lactate production reduced 6 h post-HIR in mice treated with 5-ALA/CHIL3 (Figure 7D-F). Concomitantly, post-HIR injury was further alleviated in mice treated with 5-ALA/CHIL3 (Figure 7G-I).

## Discussion

For liver transplantation, most donor livers are harvested in ischemic conditions, followed by organ preservation and reperfusion. Significant HIR injury to the donor graft would trigger inflammatory response in the recipients, hampering recovery and repair of the transplanted liver 1, 12, 34. Major liver surgeries all involve blood flow cessation, hence HIR injury is a common clinical problem that researchers have been dealing with for years. Because in many post-operation cases patients need to take immunomodulating drugs, while excessive inflammatory response exacerbates hepatocellular injuries, research on HIR injury has been largely focused on the events of cell death and immune response 2, 4, 6. Nonetheless, the liver is the main organ for metabolism and detoxification and has good capabilities of self-repair and regeneration, while HIR first and foremost disrupts hepatic metabolic homeostasis. Thus, metabolic intervention at the early stage of HIR may be able to mitigate initial damage and subsequent inflammation, hopefully relieving the burden on immunomodulation.

In this regard, we sought inspiration from hibernating mammals as they are masters of metabolic adaptation and well known for their tolerance of various types of ischemia-reperfusion stress 35-41. From cultured hepatocyte-like cells derived from induced pluripotent stem cells of the 13-lined ground squirrels 7, 42, 43, we found that the cellular level of 5-ALA increases during cold exposure or OGD, and then decreases during rewarming or reoxygenation. We propose that 5-ALA can modulate the activity of mitochondrial Complex III, alleviate rewarming-induced mitochondrial stress and restore ATP production and redox homeostasis. In patients and mouse liver resection and regeneration models, we unveiled the correlation between 5-ALA synthesis and good liver recovery, and that 5-ALA supplements affect the expression of macrophagic chemokine receptors and M2 polarization 8. In the context of HIR, the protective role of HO-1 (HMOX1) against organ ischemia-reperfusion injury has been well studied 9, 44, 45. Indeed, we found that 5-ALA treatment could further elevate the expression of *Hmox1* in mice following HIR (Figure S1C). Nonetheless, the expression of *ALAS1* was upregulated in post-transplantation liver samples (Figure 1A), and higher levels of *ALAS1* expression and 5-ALA are associated with better liver recovery following split liver transplantation 8. These findings demonstrate the importance of upstream 5-ALA metabolic activities in protecting the liver from various types of stress and injuries. Interestingly, it has been proposed that Himalayan marmots upregulate the catabolism of unsaturated fatty acids and the expression of insulin-like growth factor binding proteins to avoid liver injury during hibernation 46. Understanding how 5-ALA metabolism and lipid metabolism are orchestrated to support the recovery of hepatic redox and energy homeostasis in hibernating species may help elucidate how superior metabolic adaptation can be achieved in mammals.

Notably, other researchers have reported successful cases in applying 5-ALA and ferrous ion to protect mice from kidney and fatty liver ischemia-reperfusion injury, and induce permanent cardiac allograft acceptance by differentially regulating T cells and dendritic cells 47-49. Here we demonstrated that 5-ALA treatment at the ischemic phase alone was effective in mitigating HIR injury in normal mice. It would be exciting to assess whether supplementing 5-ALA in the organ procurement, preservation and transplantation stages would significantly restrict cold and warm ischemia-reperfusion injury in donor livers. Importantly, here we show that during HIR 5-ALA represses the RelA-CX3CR1 axis and promotes macrophagic M2-like polarization. Thus, inhibiting the RelA-CX3CR1 pathway may also be considered to attenuate HIR injury. However, liver surgery-associated HIR injury varies among patients and is difficult to be distinguished from other surgery-related injuries, while RelA-CX3CR1 inhibitory drugs may interfere with the effects of other immunomodulating drugs patients may need to take. In contrast, 5-ALA and CHIL3/CHI3L1 are naturally produced in our body, and supplemented 5-ALA or CHIL3/CHI3L1 did not manifest a long-lasting effect (Figure S5E-G). Nonetheless, caution should be given to patients with lead poisoning or defects in 5-ALA-heme metabolism, as 5-ALA supplements may cause porphyria 50-53 in these patients.

Here we found that 5-ALA may attenuate HIR injury via its complex modulation on post-HIR lipid metabolic activities, and the CX3CR1-CHIL3/CHI3L1 axis serves as an unexpected downstream effector in this context. Elevated levels of CHIL3/CHI3L1 have been reported in various disease models 54, 55. In mouse stroke models, KO of *Chi3l1* inactivates microglial M2 polarization, enhances neuroinflammation and exacerbates ischemia-reperfusion injury 56, which is in line with our findings in mouse HIR. Moreover, here we provide evidence to demonstrate unexpected functions of CHIL3/CHI3L1 proteins following their internalization by HIR- or OGD/R-stressed hepatocytes. Apparently, internalized CHIL3/CHI3L1 proteins trigger hepatocellular metabolic responses, facilitating lipid metabolism and ATP production whilst repressing mitochondrial oxidative phosphorylation (Figure 6 and S6). As high-dose treatment with 5-ALA may not be applicable to patients with acute hepatic porphyria or lead poisoning 57, additional treatment with CHIL3/CHI3L1 could provide extra efficacy to mitigate HIR injury.

## Materials and Methods

### Patient samples

All liver samples and serum samples used in this study were collected in the Liver Transplantation Center of the Third Affiliated Hospital of Sun-Yat Sen University (Guangzhou, China). All patients provided written informed consent. The use of human samples complied with the Declaration of Helsinki and was approved by the Institutional Ethics Committee of the Third Affiliated Hospital of Sun Yat-Sen University ([2022]02-253-01).

### Animals

*Wild type* (*WT*) male C57BL/6J and* Cx3cr1* KO mice (8-10 weeks old) were used in this study. *WT* mice were purchased from the Zhuhai BesTest Bio-Tech Company (Guangdong, China). *Cx3cr1* KO mice were purchased from Jackson Laboratories (Bar Harbor, ME, USA) and were backcrossed to the C57BL/6J background for more than 10 generations. The mice were housed in a specific pathogen-free environment with a controlled temperature of 23 ± 1.5°C and relative humidity of 70 ± 20%. Experimental protocols and animal care methods were reviewed and were approved by the Institutional Animal Care and Use Committee of Jennio Biotech Co., Ltd (JENNIO-IACUC-2022-A006, Guangzhou, China). and performed according to their guidelines.

### Mouse HIR injury model

All mice underwent sham surgery or hepatic IR surgery. The standard mouse 70% liver IR injury model was established as previously described 58. In brief, after anesthesia with pentobarbital sodium (50 mg/kg i.p.), mice were subjected to laparotomy, and then the portal vein, hepatic artery, and bile duct were clamped with an atraumatic vascular clip blocking the blood supply to the median and left lateral lobes of the liver. After 90 minutes of ischemia, the clip was removed, and the blood supply was restored following. After 6 or 24 hours of reperfusion, the blood was drawn from the hearts under isoflurane anesthesia, and liver tissues were collected. The sham mice were only subjected to laparotomy. For 5-ALA treatment, the dose of 5-ALA has been optimized *in vivo* and *in vitro*, and taken consideration from previous works 46.

### Measurement of porphobilinogen (PBG) and ALAD activity

Liver tissue were weighted and homogenized in lysis buffer 7. Then 100 μL sample and PBG standard were mixed with 100 μL fresh Modified Ehrlich reagent and incubated for 10 minutes at 37 °C. Concentration of PBG was determined absorbance at 555 nm.

The activity of ALAD was determined using a colorimetric assay 59. The liver tissues were collected in Tris-acetate buffer and homogenized. 12 µl of 100 mM 5-ALA was added into lysis buffer and incubated at 37 °C. The reaction was stopped by HgCl_2_ and the concentration of PBG was determined as above. The ALAD activity is calculated as the synthesis rate of PBG.

### Measurement of hepatocellular damage

The serum was isolated from cardiac blood sample. Alanine aminotransferase (ALT), aspartate aminotransferase (AST) and lactate dehydrogenase (LDH) levels in serum were measured by using a 7180 Biochemical Analyzer (Hitachi, Japan).

### Hematoxylin and Eosin Staining

The degree of liver injury was also assessed based on the pathology analysis. The fixed liver tissues were embedded in paraffin wax, and then 4 μm-thick liver sections were cut and stained with hematoxylin and eosin (H&E). The morphology results were evaluated by a pathologist who was blinded to the experimental groups. The histological severity of HIR injury was graded using Suzuki's method.

### Isolation of primary mouse hepatocytes and macrophages

Primary mouse hepatocytes and macrophages was isolated via a two-step collagenase perfusion technique, according to previous studies 60. In brief, mouse was anesthetized by inhalation of isoflurane. Portal vein was canulated and perfused with 0.5 mM EGTA in Hank's balanced salt solution (HBSS, GE, USA) without calcium and magnesium to flush intrahepatic blood. Then, the liver was perfused with 0.4 mg/mL type IV collagenase (Sigma-Aldrich, USA) and digested for 7-8 min. Next, the liver was harvested and torn apart. Cells suspension was collected and filtered through 70 μm membrane. Filtered cells were centrifuged at 4 °C for 3 min at 50 g. The supernatant was collected for the isolation of macrophages while the sediment was suspended for the isolation of hepatocytes. 50% and 90% Percoll solutions were used for the separation of macrophages and hepatocytes. Macrophages were allowed to attach for 30 min and hepatocytes were allowed to attach for 4 h. Subsequently, the medium was replaced and cells were allowed to grow overnight before further experiments.

### Mouse peritoneal macrophages (PMs) isolation and cell culture

Attach 25-G needle and inject 2 ml of 3% Brewer thioglycollate medium per mouse into the peritoneal cavity. Allow inflammatory response to proceed for 4 days, then euthanize mice by rapid cervical dislocation. Soak the abdomen of each mouse with 70% alcohol and then make a small incision along the mid-line with sterile scissors. Retract the abdominal skin manually to expose the intact peritoneal wall. Inject 5 ml of the cold harvest medium into each mouse to cause tenting of the peritoneal wall. dispense peritoneal fluid into a 50-ml conical polypropylene centrifuge tube on ice. Centrifuge the peritoneal exudate cells (PEC) in a refrigerated centrifuge 10 min at 4 °C 400 g (∼1000 rpm in Eppendorf 5810R, Germany). Discard supernatant and resuspend cell pellet in cold DMEM/F12-10 by gently tapping the bottom of the tube and pipetting up and down.

### Oxygen-glucose deprivation and reoxygenation (OGD/R) model

For cell cultures, all cells were incubated at 37 °C in a humidified atmosphere with 5% CO_2_. For oxygen-glucose deprivation experiments, normal culture medium was replaced with low-glucose Dulbecco's Modified Eagle Medium (C11995500BT, Thermo Fisher Scientific, MA, USA) supplemented with 1% FBS (P30-3302, PAN, Germany). Cells in this group were incubated at room temperature for 10 minutes before being exposed to a hypoxic environment with 5% CO_2_, 1% O_2_, and 94% N_2_ in a 37 °C incubator for the specified duration. The hypoxic medium of the cultures in the reoxygenation group was replaced with the normal culture medium, and then the cells were cultured at normal conditions for the designated time.

### TUNEL staining

The liver apoptosis degree was detected through terminal deoxynucleotidyl transferase dUTP nick-end labeling (TUNEL) assay using a commercial detection kit (11684795910, Roche Diagnostics, IN, USA) according to the manufacturer's instructions. Briefly, the TUNEL reaction mixture was mixed with the enzyme and label solutions, then each tissue section was incubated with the reaction mixture for 90 minutes at 37 °C in the dark. After that, the sections were washed twice with PBS and incubated with DAPI (D1306, Invitrogen, MA, USA) for 1 minute at room temperature. The tissue sections were photographed under a fluorescence microscope (LSM880, Zeiss, Germany). Five fields were randomly selected from each section for assessment of the necrotic area, and the percentage of apoptotic cells was analyzed by an observer who was blinded to the experimental groups.

### Cell imaging and analysis of mitochondrial ROS

MitoNeoD (563761, MedKoo Biosciences, NC, USA) is a novel mitochondrion targeted probe for superoxide production. For living cells, Cells after 4 hours OGD were incubated in normal medium containing 2.5 μM MitoNeoD for 20 minutes in the dark at room temperature. The live cell was imaged under LSM880 system (Zeiss, Germany). At least 5 different well-focused images of cells per experiment were measured. For mouse liver, MitoNeoD (10 mg/kg, intraportal infusion) was injected into mouse liver 20 minutes before ischemia and reperfusion.

### Western Blots

Electrophoresis on 10% sodium dodecyl sulfate-polyacrylamide gels were used to separate the cellular proteins, which were then transferred to polyvinylidene difluoride membranes for immunoblotting. The membranes were blocked for 1 hour at room temperature with 5 % skimmed milk in tris-buffered saline (TBS), supplemented with 0.1% Tween 20. The blots were incubated overnight with diluted primary antibodies, and then incubated for 1 h with a secondary antibody at room temperature. Subsequently, the membranes were again washed three times for 10 min each, and the antigen-antibody complexes were visualized using Mini-PROTEAN Tetra system (Bio-Rad, CA, USA). The specific protein bands were imaged and analyzed on ChemiDoc Imaging System (Bio-Rad, CA, USA).

### Quantitative Real-time PCR

Cellular RNA was isolated using an RNA isolation reagent, and the concentrations obtained were quantified by measuring the absorbance of each sample at 260 nm. Quantities of 6 μg total RNA were used for reverse transcription, and then the expression of inflammation-related factors and macrophage polarization markers both in liver tissue and isolated macrophages were quantified using real-time PCR (RT-PCR). Table S2 illustrates primers used for RT-PCR. ACTB was used as the reference gene.

### Immunofluorescence staining

To perform immunofluorescence staining, 8 μm-thick frozen liver tissue sections were rinsed, blocked and incubated with primary antibodies overnight at 4 °C. The sections were then rinsed twice, incubated with the secondary antibody for 1 hour at room temperature. The sections were observed under the fluorescence microscope (Zeiss, Germany). The secondary antibody was obtained from Invitrogen (DAKO, CA, USA). the immunofluorescence was analyzed by Image J (NIH, MD, USA).

### BODIPY staining

Liver sections and cell samples were incubated with BODIPY (Invitrogen, MA, USA) for 30 minutes in the dark at room temperature, and mounted using mounting medium containing DAPI (D3922, Invitrogen, MA, USA).

### ELISA

Level of IL-1β (EMC001b, Neobioscience), IL-6 (EMC004, Neobioscience), IL-10 (EMC005, Neobioscience) and TNF-α (EMC102a, Neobioscience) in cell supernatants were measured. And CHIL3 levels in serum were measured using an ELISA kit (ELM-YM1, RayBio, GA, USA). According to the manufacturer's instructions. Added samples and standard into strips and incubate for 2.5 hours at room temperature with gentle shaking. Discard the solution and washed. Added 100 µl of prepared biotinylated antibody for 1 hour. Then reacted with prepared Streptavidin solution. TMB One-Step Substrate Reagent used. 45 minutes after chromogenic reaction, stop reaction was added and each well read at 450 nm immediately.

### Indirect calorimetry

The mice were housed individually in metabolic chambers for 2 days to minimize the stress of housing change. The mice were then measured in the calorimetry chambers for another 2 days with a high-resolution recording at 5 minutes intervals (CLAMS HC Comprehensive Lab Animal Monitoring System, Columbus Instruments, OH, USA). The mice with or without 5-ALA (30 mg/kg) for HIR injury were measured in real time. The data was analysis by Oxymax (Columbus Instruments, OH, USA) and Prism (Graphpad, CA, USA).

### Seahorse assay

An XF96 seahorse (Seahorse Bioscience, MA, USA) was applied to detect the ATP rate of PMs. A total of 20,000 cells/well which had been transferred and seeded into the Seahorse XF96 culturing plates within medium. Wells divided into different treatment groups. Next day, cells were gently washed once in PBS and then cultured for one hour at 37 °C in Seahorse incubation medium. To ensure accurate detection of extracellular pH, cells were cultured in a CO_2_-free incubator. The detection of OCR and extracellular acidification rate (ECAR) were performed at baseline and following sequential injections of oligomycin (2.5 μM), rotenone (1μM) and antimycin-A (1μM). All data were automatically calculated by the Seahorse XF96 software (Wave, Seahorse Bioscience, MA, USA).

### Flow cytometry

Mouse livers were perfused with HBSS via the portal vein and followed by 0.04% collagenase IV (C5138, Sigma, MO, USA). Perfused livers were dissected and teased upon 70-μm cell strainers, then suspended in 40 mL DMEM supplemented with 10% FBS. Non-parenchymal cells (NPCs) were separated from hepatocytes by centrifugation at 50 × g for 2 minutes three times. NPCs were plated in cell culture dishes in DMEM supplemented with 10% FBS, 10 mM HEPES, 2 mM GlutaMax, 100 U/mL penicillin, and 100 mg/mL streptomycin for 15 minutes at 37°C, then the non-adherent cells were removed. The CD45^+^F4/80^+^ macrophages adherent cells were used for Flow cytometry. Antibodies used were anti-CD45-PerCP/Cyanine5.5 (103131, Biolegend, CA, USA), anti-CD11b-BV510 (101263, Biolegend, CA, USA), anti-F4/80-APC (123116, Biolegend, CA, USA), anti-CD206-PE (141706, Biolegend, CA, USA) and anti-CD86-BV605 (105037, Biolegend, CA, USA). Cells stained with corresponding isotype control antibodies were used as controls. Data were analyzed using CytExpert software (Beckman, CA, USA).

### Cell viability analysis

Cell Counting Kit-8 (A311, Vazyme, China) was used to determine the number of viable cells according to the manufacturer's protocol. A cell suspension containing 1×10^4^ cells/100 µL medium was added to each well in a 96-well plate. 10 µL of CCK8 assay reagent was added into wells exposed to OGD/R experiment or not. The cell viability was measured by the increase in absorption at 600 nm. The fold changes were calculated using baseline values of control cells.

### Live cell imaging and analysis of mitochondrial membrane potential (Δψm)

The change of Δψm was measured using the cell-permeable and positively charged dye tetramethylrhodamine ethyl ester (TMRE, HY-D0985A, MCE, NJ, USA), which accumulates in active mitochondria characterized by a negative net charge, whereas depolarized mitochondria do not retain the dye. Prior to analysis, cells were incubated in fresh medium supplemented with 50 nM TMRE for 10 minutes at 37 °C. Then the cells were imaged under LSM880 system (Zeiss, Germany). After 4 hours of OGD and 1 hour reoxygenation, the TMRE signal were recorded under LSM880 system. At least 5 different well-focused images per experiment were measured. The data are analysis by Image J (NIH, MD, USA).

### NAD^+^/NADH, NADP^+^/NADPH and ATP assays

The NAD^+^/NADH Assay kit (K347-100, BioVison, CA, USA) and the NADP^+^/NADPH Assay kit (K347-100, BioVison, CA, USA) were used according to the manufacturer's instructions. Briefly, 2×10^5^ cells were extracted in 100 μL of ice-cold Extraction Buffer. To measure total NADH or NADPH, 50 μL of lysates were transferred to PCR tubes; For measure NADH or NADPH, another 50 μL of lysates were transferred to PCR tubes and were incubated at 60 °C for 15 minutes. Under these conditions, all NAD^+^ and NADP^+^ will be decomposed while the NADH and NADPH will still be intact. Then the 50 μL of samples were transferred to wells and were incubated in the Reaction Mix. The luminescence levels were measured using a plate reader. ATP was detected using a Colorimetric assay kit (Nanjing Jiancheng Bioengineering Institute, China) with a 636 nm luminescence measurement. The data are expressed as relative fluorescence levels adjusted for protein levels, with the mean values measured at 37 °C set to 1.

### Measurement of cellular lactate production

L-lactate was measured in cells and liver tissues using the L-lactate Assay kit (BC2230, Solarbio, China) according to the manufacturer's instructions. All samples were deproteinized and reacted with lactate dehydrogenase to form NADH and H^+^. H^+^ is passed to PMS and reacted with MTT to produce purple material with characteristic absorption peak at 570 nm. Protein concentration in samples before deproteinization was measured for normalization.

### Mitochondrial complex III, complex IV, complex V activity

For complex III activity was evaluated spectrophotometrically at 37 °C by monitoring the increase in absorbance at 550 nm of cytochrome c. Briefly, complex I inhibitor (2 μg of rotenone, R8875, Sigma), complex II inhibitor (5 mM decylubiquinone, D7911, Sigma), complex IV inhibitor (5 mM NaN_3_, S2002, Sigma) and 60 μM cytochrome c (C2867, Sigma) and 0.1 mg of whole-cell lysate were mixed. The reaction was stopped by the addition of complex III inhibitor (2 μg antimycin A, A8674, Sigma) and measured absorbance at 550 nm. One unit was defined as nmol· min -1· μg -1 of protein. For complex IV and complex V activity were determined by determined the mitochondrial complex IV activity assay kit (BC0940, Acmec, China) and mitochondrial complex V activity assay kit (BC1445, Solarbio, China) according to the manufacturer's instructions. Briefly, cells were homogenized in an extraction buffer. The homogenate was centrifuged to get mitochondrial fraction. The mitochondrial fraction was incubated in an assay solution and the OD was read at 550 nm for complex IV, 660 nm for complex V. One unit of complex IV activity was defined as the oxidization of 1.0 nmol of reduced cytochrome c per min in 1 mg of protein. One unit of complex V activity was defined as the oxidization of 1.0 nmol of increased Pi per min in 1 mg of protein. The fold changes were calculated using baseline values of control cells as a reference.

### RNA sequencing

Total RNA was extracted from frozen mouse liver tissues or L-02 samples using TRIzol (15596026, Invitrogen, USA) according to the manufacturer's instructions. After mRNA quantification and qualification, we used NEBNext® UltraTM RNA Library Prep Kit (E7775, NEB, MA, USA) for sequencing libraries preparation, which following manufacturer's recommendations. Then the prepared RNA-seq libraries were purified and library quality was assessed using an Agilent 2100 system (Illumina, CA, USA). The clustering of the index-coded samples was performed, after that, the library preparations were sequenced on an Illumina Novaseq platform and 150 bp paired-end reads were generated.

### RNA sequencing data analysis

Raw data (raw reads) of fastq format were firstly processed through Trimmomatic v0.39 61. At the same time, Q20, Q30 and GC content the clean data were calculated. All the downstream analyses were based on the clean data with high quality. Reference genome and gene model annotation files were downloaded from Ensembl website directly. Index of the reference genome was built using Hisat2 v2.2.1 and clean reads were aligned to the reference genome using Hisat2 v2.2.1 to generate a .sam or .bam files containing the alignments 62. The gene expression level quantification was performed separately for each sample using the featureCounts v2.0.1 software 63. Then results of all samples were combined to obtain the expression matrix of all samples. Given the pair-wise design of the experiments, differentially expressed genes were identified using DESeq2 v1.26.0 64. P values from differential expression tests were adjusted using the Benjamini-Hochberg procedure for multiple hypothesis testing, and genes with an adjusted *P*-value < 0.1 and |log_2_(foldchange)|> 0.3 were identified as differentially expressed. Pathway analysis were performed using the Metascape web interface 65.

### Statistical analysis

Data were analyzed using one-way ANOVA and Student's *t*-test, and statistical significance was presented as *P < 0.05, **P < 0.01, ***P < 0.001 in all figures.

## Supplementary Material

Supplementary figures and tables.

## Abbreviations

5-ALA: 5-Aminolevulinate
ALAD: 5-Aminolevulinate dehydratase
ALAS1: 5-aminolevulinate synthase 1
ALT: alanine transaminase
AST: aspartate transaminase
ATP: adenosine triphosphate
CHI3L1: chitinase-3-like protein 1
CHIL3: chitinase-like 3
CX3CR1: CX3C motif chemokine receptor 1
DAPI: 4,6-diamidino-2-phenylindole
DEGs: differentially expressed genes
ECAR: extracellular acidification rate
HIR: hepatic ischemia-reperfusion
HO-1: heme oxygenase-1
IL-4: interleukin-4
LDH: lactate dehydrogenase
LPS: lipopolysaccharide
OCR: oxygen consumption rates
OGD/R: oxygen-glucose deprivation and reoxygenation
OXPHOS: mitochondrial oxidative phosphorylation
PBG: porphobilinogen
PMs: peritoneal macrophages
ROS: reactive oxygen species
TMRE: tetramethylrhodamine, ethyl ester
UROS: uroporphyrinogen-III synthase
UROD: uroporphyrinogen decarboxylase
