# Supplementary material for: 5-aminolevulinate and CHIL3/CHI3L1 treatment amid ischemia aids liver metabolism and reduces ischemia-reperfusion injury
Source: Theranostics. 2023 Aug 28;13(14):4802–20. doi: 10.7150/thno.83163 (PMC10526663; doi:10.7150/thno.83163)
Supplement: Supplementary file 1 — Supplementary figures and tables. [file thnov13p4802s1.pdf]

Supplemental Figures and legends

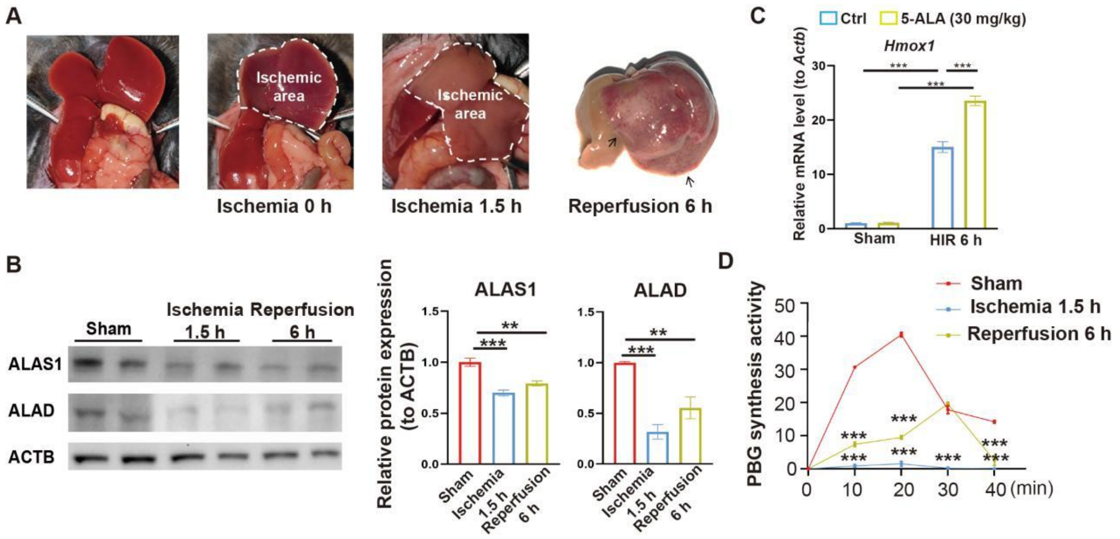

Figure S1. 5-ALA metabolic pathway in mouse HIR model. Related to Figure 1.

(A) Representative images demonstrating the mouse HIR operation (arrows denote hepatic areas with HIR injury).

(B) Representative western blots on the hepatic content of key enzymes ALAS1 and ALAD in the 5-ALA metabolic pathway from mice at indicated conditions (n = 4). Their signal intensity is normalized to that of ACTB.

(C) Relative mRNA levels of *Hmox1* in mouse livers at indicated conditions normalized to that of *Actb* (n = 6).

(D) Activity of ALAD at indicated conditions (n = 3).

Results are presented as mean ± SEM. *$P < 0.05$, **$P < 0.01$, ***$P < 0.001$ by one-way ANOVA (B), (C) and (D).

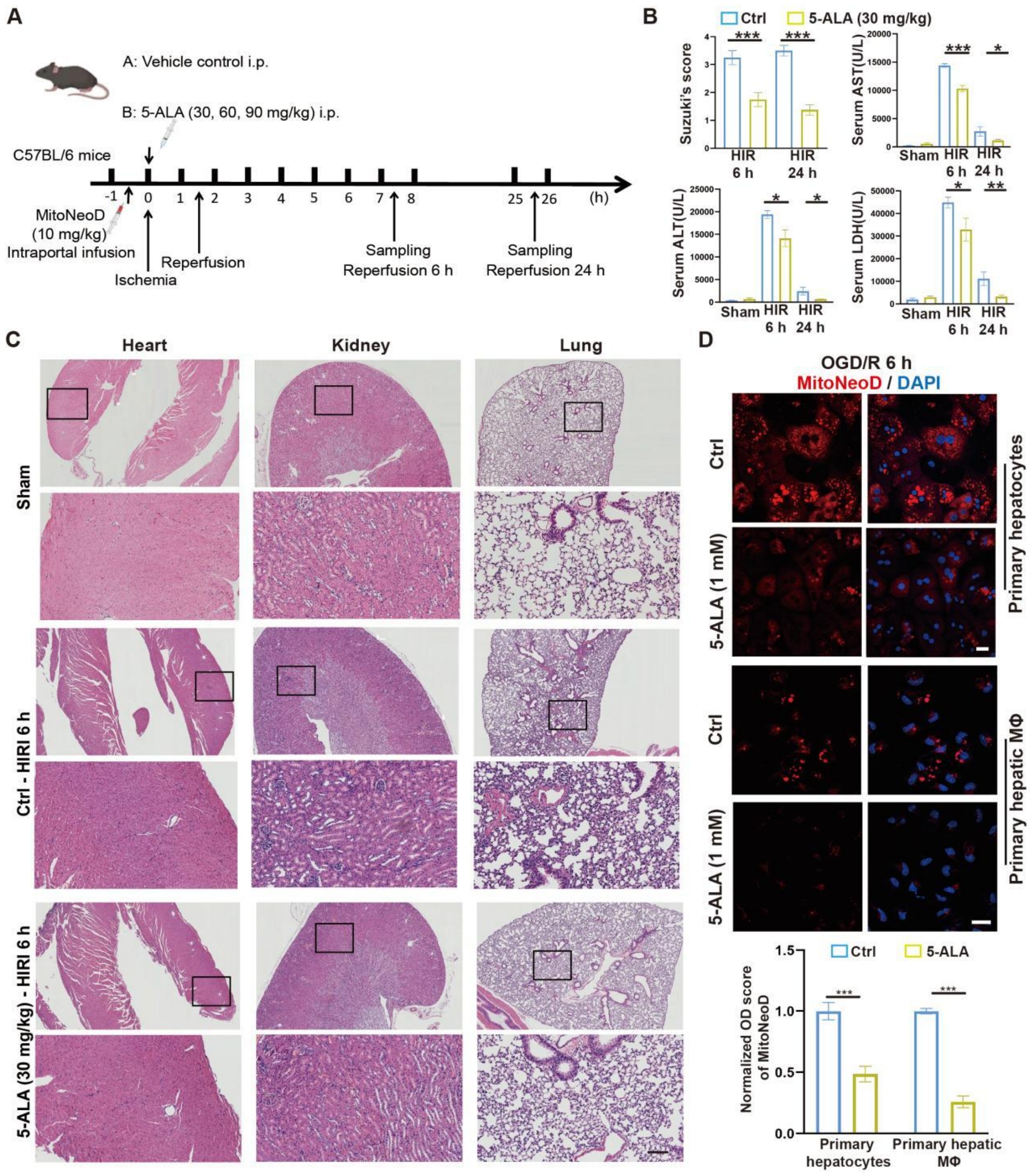

Figure S2. Supplemented 5-ALA relieves HIR-induced injury and mitochondrial ROS overproduction. Related to Figure 2.

(A) Outline of the mouse HIR experimental procedures and conditions.

(B) Liver injury indicators Suzuki's score, AST, ALT and LDH levels in mice at indicated conditions (n = 8 per group).

(C) Representative H&E staining of heart, kidney and lung sections from mice at indicated conditions. No overt abnormality was found in the 5-ALA treatment group (scale bar = 0.1 mm).

(D) MitoNeoD (red) and DAPI (blue) staining to evaluate mitochondrial ROS overproduction in mouse primary hepatocytes and hepatic macrophages at annotated conditions (n = 5 experiments, scale bar = 10 μm).

Results are presented as mean ± SEM. *$P < 0.05$, **$P < 0.01$, ***$P < 0.001$ by two-tailed Student's *t* tests (D) or one-way ANOVA (B).

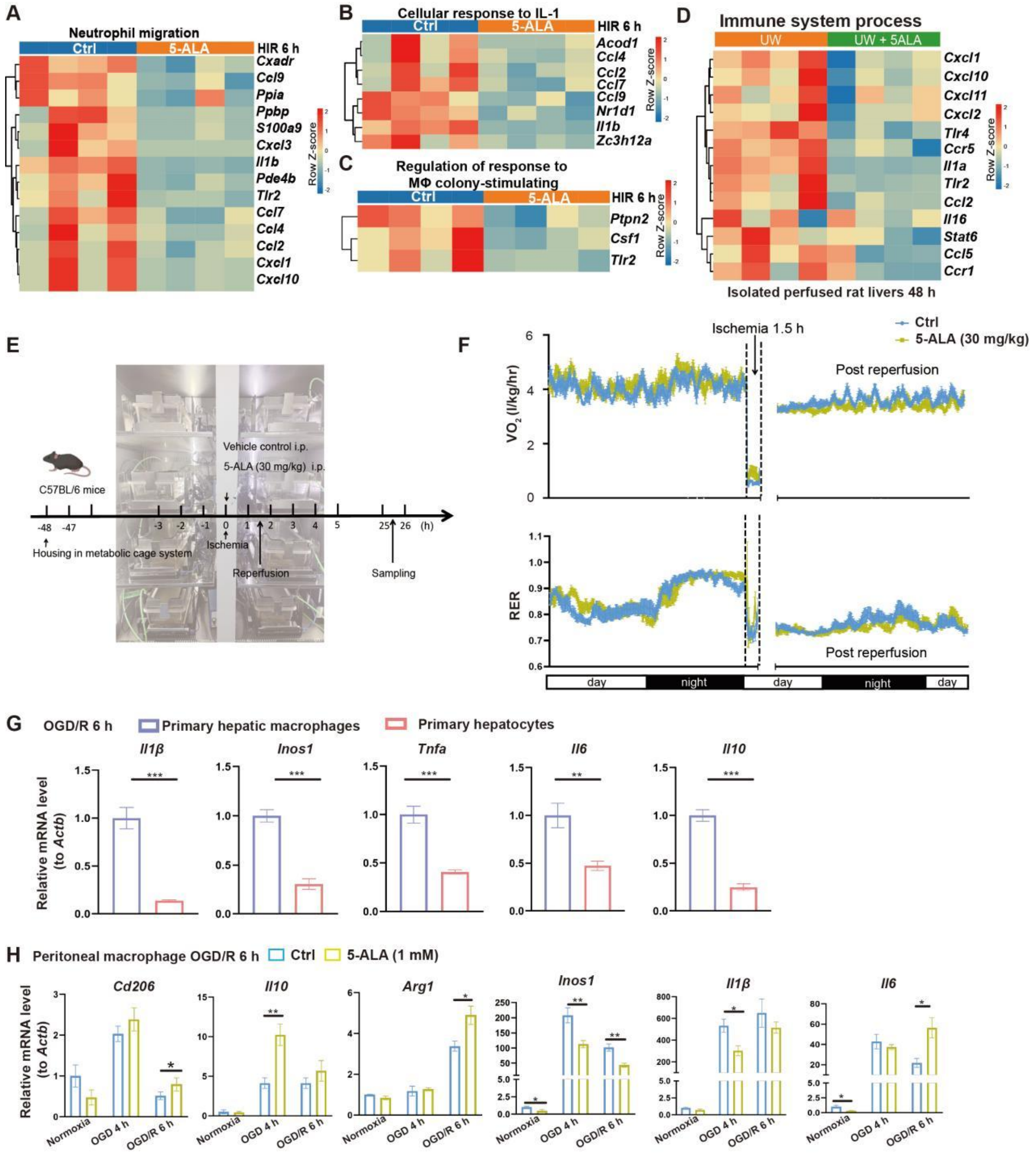

Figure S3. 5-ALA modulates mouse hepatic metabolism and immune responses following HIR. Related to Figure 3.

(A) Heatmap of differentially expressed genes (DEGs) related to neutrophil migration (GO:1990266) in mouse liver samples at annotated conditions (n = 4 per group).

(B) Heatmap of DEGs related to cellular response to IL-1 (GO:0071347) in mouse liver samples at indicated conditions (n = 4 per group).

(C) Heatmap of DEGs related to regulation of response to macrophage colony-stimulating factor (GO:1903969) in mouse liver samples at indicated conditions (n = 4 per group). Red, high relative expression; blue, low relative expression.

(D) Heatmap of DEGs related to immune system process (GO:0002376) in isolated perfused rat liver at indicated conditions (n = 4). Red, high relative expression; blue, low relative expression.

(E) Outline of the HIR experimental conditions and setup of the mouse metabolic cage system.

(F) Representative results of oxygen consumption rates (OCR, VO_2) and respiratory exchange ratio (RER) in control and 5-ALA-treated mice measured as indicated with the mouse metabolic cage system. Values normalized to the body weight of each mouse (n = 6 per group).

(G) Relative mRNA expression of inflammatory cytokines (*Il1β, Inos1, Tnfa, Il6* and *Il10*) of in mouse primary macrophages and hepatocytes 6 h after OGD/R (n = 6 per group). The mRNA level of each gene is normalized to that of *Actb*.

(H) Relative mRNA expression of macrophagic M1-enriched genes (*Cd206, Il10, Arg1*) and M2-enriched genes (*Inos1, Il1β, Il6*) in mouse peritoneal macrophage cultures at indicated conditions (n = 6 per group). The mRNA level of each gene is normalized to that of *Actb*.

Results are presented as mean ± SEM. *$P < 0.05$, **$P < 0.01$, ***$P < 0.001$ by two-tailed Student's *t* tests (G) or one-way ANOVA (H).

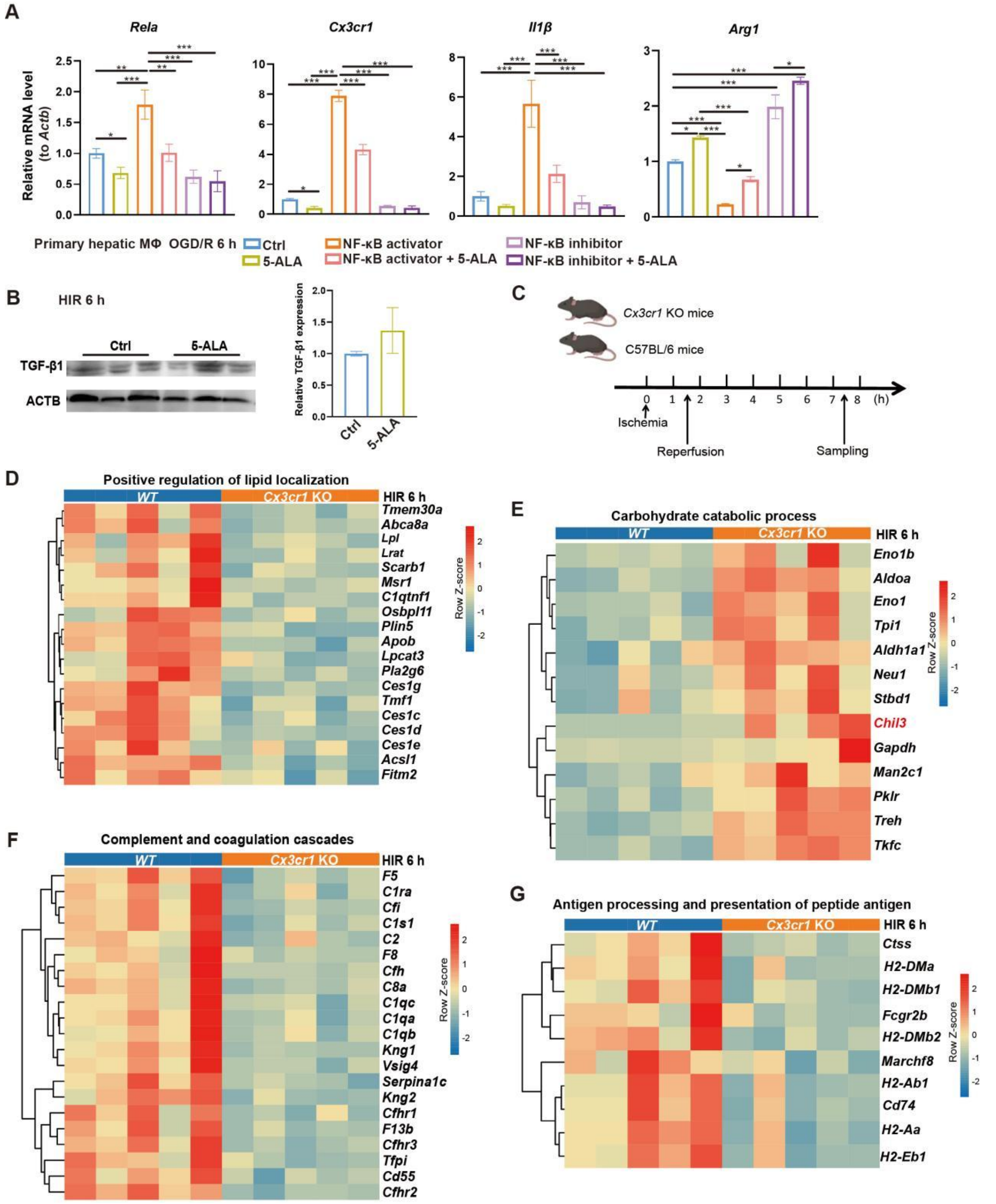

Figure S4. CX3CR1 deficiency profoundly affects hepatic metabolism and immune responses as downstream effects of 5-ALA treatment during mouse HIR. Related to Figure 4.

(A) Relative mRNA expression of *Rela, Cx3cr1, Il1β* and *Arg1* in mouse primary hepatic macrophages at indicated conditions (NF-κB activator: 8 μg/mL betulinic acid,

NF-κB inhibitor: 5 μM BAY-11-7082, 5-ALA: 1 mM, n = 6 per group). The mRNA level of each gene is normalized to that of *Actb*.

(B) Representative western blots of TGF-β1 in mouse livers at indicated conditions (n = 3). Their signal intensity is normalized to that of ACTB.

(C) Outline of the HIR experimental procedures in *WT* and *Cx3cr1* KO mice.

(D) Heatmap of expressed gene related to positive regulation of lipid localization (GO:1905954) differentially regulated between *WT* and *Cx3cr1* KO mouse liver 6 h after HIR (n = 5 per group). Red, high relative expression; blue, low relative expression.

(E) Heatmap of expressed gene related to carbohydrate catabolic process (GO:0016052) differentially regulated between *WT* and *Cx3cr1* KO mouse liver 6 h after HIR (n = 5 per group). Red, high relative expression; blue, low relative expression.

(F) Heatmap of expressed genes related to complement and coagulation cascades (MMU04610) differentially regulated between *WT* and *Cx3cr1* KO mouse liver 6 h after HIR (n = 5 per group). Red, high relative expression; blue, low relative expression.

(G) Heatmap of expressed genes related to antigen processing and presentation of peptide antigen (GO:0002495) differentially regulated between *WT* and *Cx3cr1* KO mouse liver 6 h after HIR (n = 5 per group). Red, high relative expression; blue, low relative expression.

Results are presented as mean ± SEM. *$P < 0.05$, **$P < 0.01$, ***$P < 0.001$ by two-tailed Student's *t* tests (B) or one-way ANOVA (A).

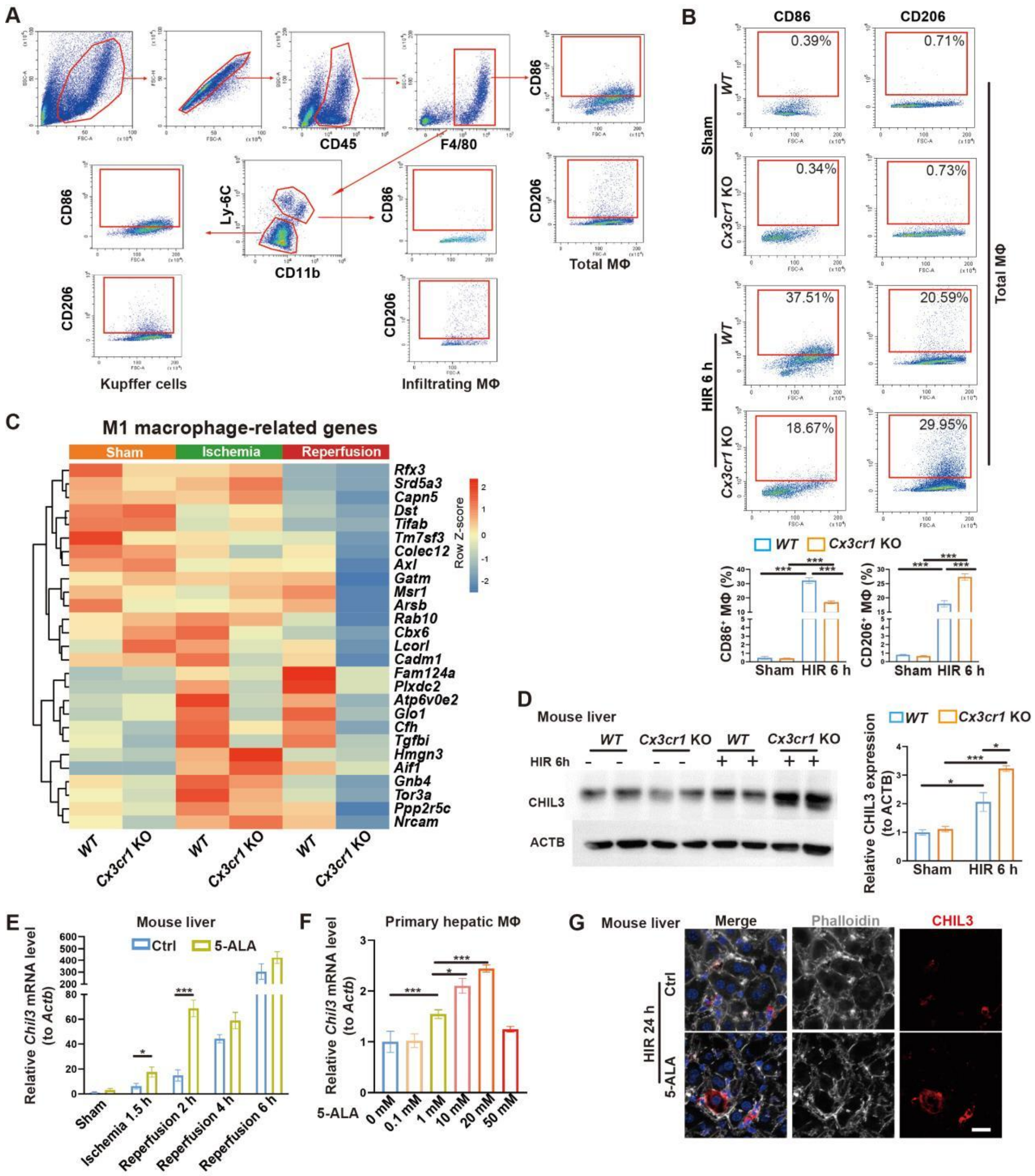

Figure S5. M2 macrophage-enriched CHIL3/CHI3L1 functions as a downstream effector of the 5-ALA-CX3CR1 axis following HIR. Related to Figure 5.

(A) Gating strategy for flow cytometry analysis of intrahepatic immune cells in Figure 5A-B.

(B) Up: flow cytometry analysis of M1 ($CD45^+F4/80^+CD86^+$) and M2 ($CD45^+F4/80^+CD206^+$) macrophages in *WT* and *Cx3cr1* KO mouse livers at indicated conditions; down: percentage of M1 ($CD45^+F4/80^+CD86h^+$) and M2

($CD45^+F4/80^+CD206^+$) macrophages in *WT* and *Cx3cr1* KO mouse livers at indicated conditions (n = 6 for sham, n = 6 for HIR 6 h).

(C) Heatmap of expressed M1-enriched genes in *WT* and *Cx3cr1* KO mouse liver samples at indicated conditions (n = 5). Red, high relative expression; blue, low relative expression.

(D) Representative western blots of hepatic CHIL3 proteins in *WT* and *Cx3cr1* KO mouse liver samples at indicated conditions. The CHIL3 signal intensity is normalized to that of ACTB (n = 4 per group).

(E) Relative mRNA levels of *Chil3* normalized to that of *Actb* in mouse livers at indicated conditions (n = 6 per group).

(F) Relative mRNA levels of *Chil3* normalized to that of *Actb* in mouse primary hepatic macrophages at indicated conditions (n = 6 per group).

(G) Representative confocal images of CHIL3 (red), phalloidin (gray) and DAPI (blue) in mouse liver sections at indicated conditions (scale bar = 10 μm).

Results are presented as mean ± SEM. *$P < 0.05$, **$P < 0.01$, ***$P < 0.001$ by one-way ANOVA (B), (D), (E) and (F).

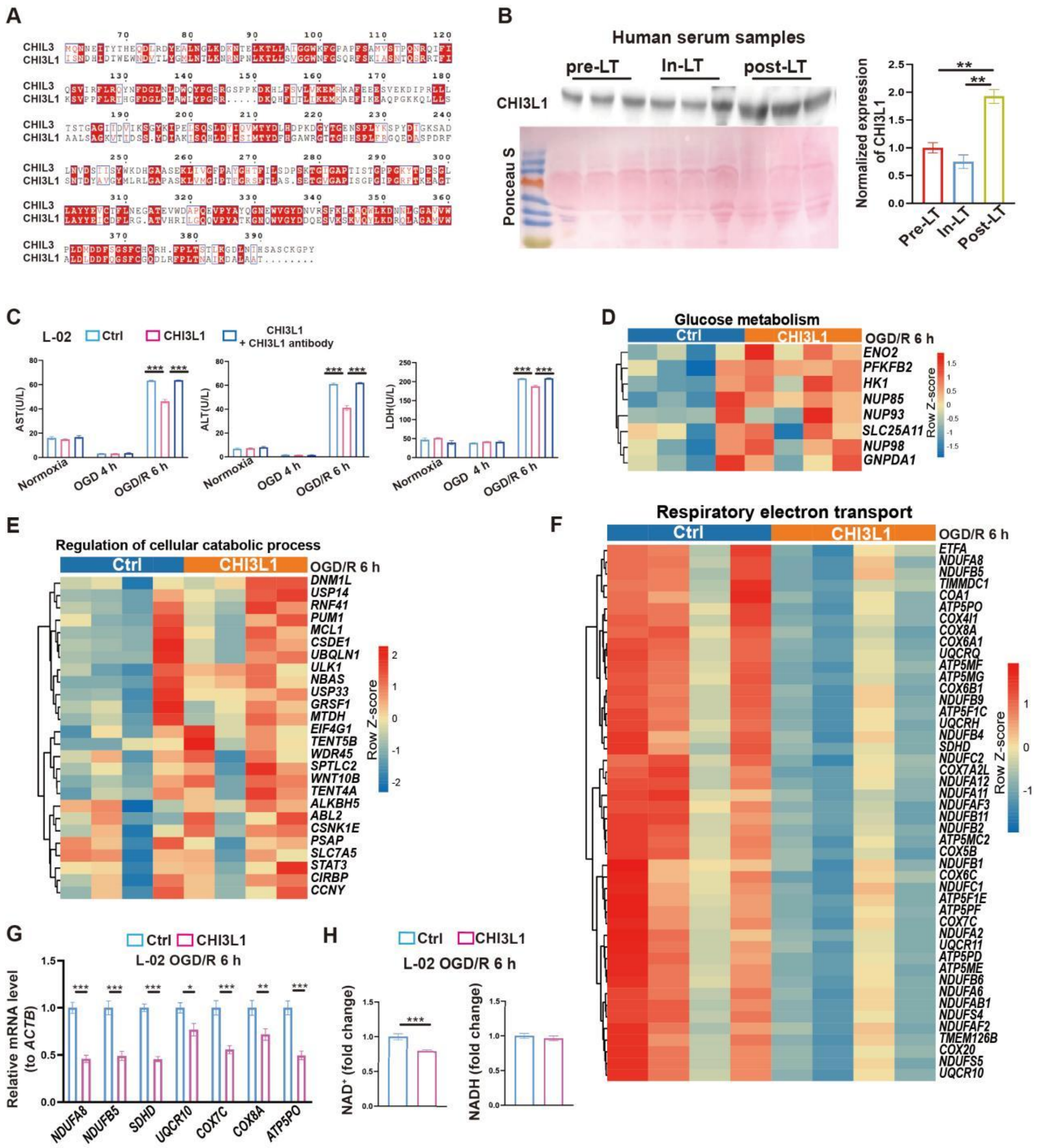

Figure S6. CHIL3/CHI3L1 affects human hepatocytic metabolism following OGD/R. Related to Figure 5 and 6.

(A) Sequence homology analysis of human CHI3L1 and mouse CHIL3 proteins.

(B) Representative western blots of CHI3L1 proteins in patients' serum samples at indicated conditions. The CHI3L1 signal intensity is normalized to the total proteins stained with Ponceau S (n = 3 per group).

(C) AST, ALT and LDH levels in medium supernatant of L-02 cultures at indicated conditions (n = 5).

(D) Heatmap of DEGs related to glucose metabolism (R-HSA-70326) in L-02 cultures at indicated conditions (n = 4 per group). Red, high relative expression; blue, low relative expression.

(E) Heatmap of DEGs related to regulation of cellular catabolic process (GO:0031329) in L-02 cultures at indicated conditions (n = 4 per group). Red, high relative expression; blue, low relative expression.

(F) Heatmap of DEGs related to respiratory electron transport (R-HSA-163200) in L-02 cultures at indicated conditions (n = 4 per group). Red, high relative expression; blue, low relative expression.

(G) Relative mRNA expression of selected genes related to the mitochondrial respiratory electron transport (*NDUFA8, NDUFB5, SDHD, UQCR10, COX7C, COX8A* and *ATP5PO*) in L-02 cultures at indicated conditions (n = 12). The mRNA level of each gene is normalized to that of *ACTB*.

(H) NAD^+ and NADH levels (n = 9) in L-02 cells at annotate conditions.

Results are presented as mean ± SEM. *$P < 0.05$, **$P < 0.01$, ***$P < 0.001$ by two-tailed Student's *t* tests (G) and (H) or one-way ANOVA (B) and (C).

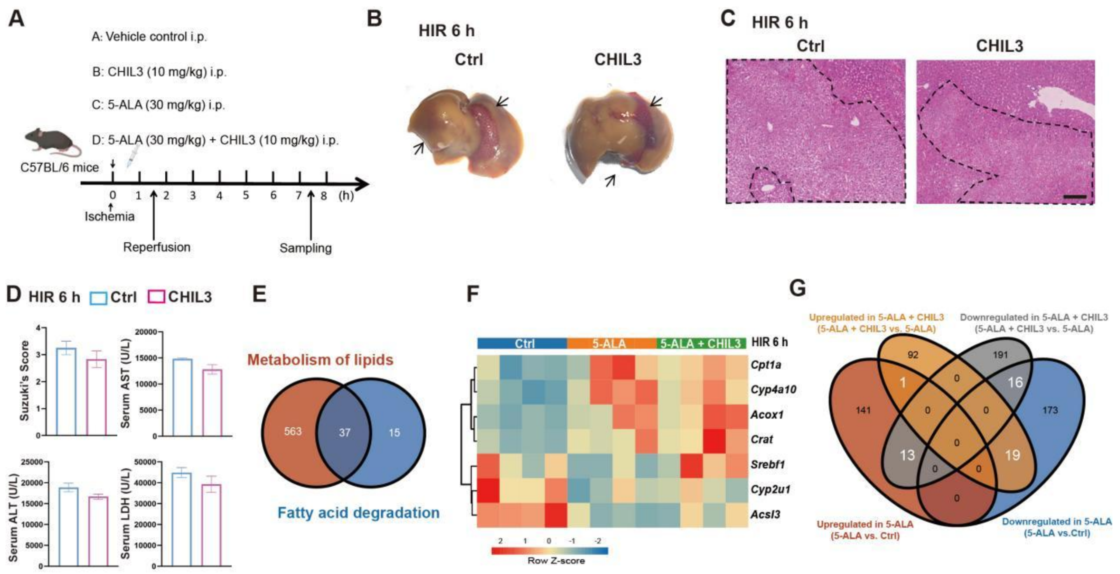

Figure S7. 5-ALA and/or CHIL3 treatments profoundly affect hepatic lipid metabolism. Related to Figure 7.

(A) Outline of the HIR experiments with mice subjected to indicated treatments.

(B) Gross appearance of mouse livers at indicated conditions (arrows denote areas of HIR injury).

(C) Representative H&E staining of mouse liver sections at indicated conditions, suggesting no overt difference between the control (ctrl) and CHIL3-treated group (areas of HIR injury are marked by broken lines; scale bar = 0.1 mm).

(D) Liver injury indicators Suzuki's score, and serum AST, ALT and LDH levels in mice with indicated conditions 6 h after HIR (n = 6 per group).

(E) Venn diagram indicating the number of overlapped genes in the metabolism of lipids (R-MMU-556833) (see Figure 3A) and fatty acid degradation (MMU00071) (see Figure 7A) pathways.

(F) Among the 37 overlapped genes from (E), heatmap of 7 genes differentially regulated by 5-ALA or a combined treatment of 5-ALA and CHIL3 6 h after HIR is

shown (n = 4 per group). Red, high relative expression; blue, low relative expression. These may be important effector genes regulated by 5-ALA and CHIL3 to facilitate liver lipid catabolism and energy production following HIR in mice. Also see Data S2.

(G) Quadruple Venn diagram indicating the numbers of overlapped DEGs in indicated comparisons. Also see Data S2.

Results are presented as mean ± SEM. *$P < 0.05$, **$P < 0.01$, ***$P < 0.001$ by two-tailed Student's *t* tests (D).

Table S1. Basic characteristics of liver transplantation donors and recipients. Related to Figure 1 and Figure S6.

	Age	Gender	Medical condition	Cold ischemia time * (min)	Time for post-operation liver biopsy (min)	Weight of liver
Donor 1	17	M	Trauma	431	67	22.1
Donor 2	59	M	Trauma	263	113	23.5
Donor 3	49	M	Trauma	357	74	21
Donor 4	27	F	Trauma	358	82	21.4
Donor 5	28	M	Trauma	381	95	23.7
Donor 6	27	M	Trauma	399	65	27.3
Donor 7	15	F	Trauma	407	132	22.5
Donor 8	55	M	Stroke	449	75	18.5
Donor 9	56	F	Encephal itis	370	79	26.6
Donor 10	32	M	Trauma	420	84	15.5
Donor 11	23	M	Trauma	500	83	26.3
Donor 12	30	M	Stroke	251	97	18.8
Donor 13	56	M	Stroke	570	102	33.2
Donor 14	50	M	Stroke	360	58	18.6
Donor 15	56	M	Stroke	340	68	20.7
Donor 16	68	M	-	230	74	22.5
Donor 17	54	F	Stroke	397	89	31
Donor 18	28	M	Stroke	330	97	22.6
Donor 19	55	M	-	429	99	15.6
Donor 20	58	M	Stroke	380	88	20.5

* Surgery-related ischemia time was typically below 30 min.

	Age	Gender	Medical condition	Liver from
Recipient 1	47	M	Liver cirrhosis	Donor 4
Recipient 2	52	M	Liver cirrhosis	Donor 10
Recipient 3	51	M	Liver cirrhosis	Donor 11

Table S2. Gene primers used for quantitative RT-PCR and genotyping.

Primers for quantitative RT-PCR related to Figure 1.

Gene	Forward Primer	Reverse Primer
ALAS1	CGCCGCTGCCCATTCTTAT	TCTGTTGGACCTTGGCCTTAG
ALAD	GCTACTTCCACCCACTACTTCG	TCAGGAACATCCGTGACAAAG
HMOX1	AAGACTGCGTTCCTGCTCAAC	AAAGCCCTACAGCAACTGTCG
UROD	ATGGAAGCGAATGGGTTGGG	GGGAGTGTAGTCTGTTTCCTCT
UROS	GCCAAGTCAGTGTATGTGGTT	GCAATCCCTTTGTCCTTGAGC
PPOX	CTGGATTCGCTCCGTTCGAG	CCCACGTAGAGGAACCTGT

Primers for quantitative RT-PCR related to Figure 4.

Gene	Forward Primer	Reverse Primer
Ccr1	GCCCTCATTTCCCCTACAA	CGGCTTTGACCTTCTTCTCA
Ccr2	TGTGATTGACAAGCACTTAGACC	TGGAGAGATACCTTCGGAACTT
Ccr5	ATGGATTTTCAAGGGTCAGTTCC	CTGAGCCGCAATTTGTTTCAC
Ccr7	CATGGACCCAGGTGTGCTTC	TCAGTATCACCAGCCCGTTG
Ccr8	TGTTTGGGACTGCGATGTGT	TGATGGCATAGACAGCGTGG
Cx3cr1	CAAGCTCACGACTGCCTTCT	TGTCCGGTTGTTCATGGAGT
Actb	AGCCATGTACGTAGCCATCC	CTCTCAGCTGTGGTGGTGAA

Primers for quantitative RT-PCR related to Figure 6.

Gene	Forward Primer	Reverse Primer
SERPINE1	ACCGCAACGTGGTTTTCTCA	TTGAATCCCATAGCTGCTTGAAT
EGFR	AGGCACGAGTAACAAGCTCAC	ATGAGGACATAACCAGCCACC
SLC2A1	GGCCAAGAGTGTGCTAAAGAA	ACAGCGTTGATGCCAGACAG
ENO1	AAAGCTGGTGCCGTTGAGAA	GGTTGTGGTAAACCTCTGCTC
HK1	GCTCTCCGATGAAACTCTCATAG	GGACCTTACGAATGTTGGCAA
PPARA	ATGGTGGACACGGAAAGCC	CGATGGATTGCGAAATCTCTTGG
ACOX1	ACTCGCAGCCAGCGTTATG	AGGGTCAGCGATGCCAAAC

Primers for quantitative RT-PCR related to Figure S1C.

Gene	Forward Primer	Reverse Primer
Hmox1	AGGTACACATCCAAGCCGAGA	CATCACCAGCTTAAAGCCTTCT

Primers for quantitative RT-PCR related to Figure S3 and S4.

Gene	Forward Primer	Reverse Primer

Inos	CCTAGTCAACTGCAAGAGAA	TTTCAGGTCACTTTGGTAGG
Il1b	CCACCTTTTGACAGTGATGA	GAGATTTGAAGCTGGATGCT
Cd206	GGTGGAAGAAGAAGTAGCCT	GAAGGGTCAGTCTGTGTTTG
Arg1	CTCCAAGCCAAAGTCCTTAGAG	AGGAGCTGTCATTAGGGACATC
Il10	TGTCAAATTCATTCATGGCCT	ATCGATTTCTCCCCTGTGAA
Il6	CCGGAGAGGAGACTTCACAGA	TCCACGATTTCCCAGAGAAC
Rela	AGGCTTCTGGGCCTTATGTG	TGCTTCTCTCGCCAGGAATAC
Tnfa	CCCTCACACTCAGATCATCTTCT	GCTACGACGTGGGCTACAG

Primers for quantitative RT-PCR related to Figure S6G.

Gene	Forward Primer	Reverse Primer
NDUFA8	CCCAACAAGGAGTTTATGCTCT	CACAGTGACGTTTTATCTGCCT
NDUFB5	AGCTGGAAGTGCGAAAATTGA	ATACCAGGGTCCATCTCCTCT
ATP5PO	ATTGAAGGTCGCTATGCCACA	GCTTTTCACTTTAATGGAACGCT

COX8A	GCCAAGATCCATTCGTTGCC	CTCTGGCCTCCTGTAGGTCT
COX7C	GGTCCGTAGGAGCCACTATGA	GTGTCTTACTACAAGGAAGGGTG
UQCR10	ATCGTGGGCGTCATGTTCTTC	ATGTGGTCGTAGATAGCGTCC
SDHD	ATTTCTTCAGGACCGACCTATCC	CAGCCTTGGAGCCAGAATG
ACTB	GTGACGTGGACATCCGCAAAGA	TGGAAGGTGGACAGCGAGGC

Primers for Genotyping related to Figure 4.

Gene	Forward Primer	Reverse Primer
Cx3cr1 wildtype	GTCTTCACGTTCGGTCTGGT	CCCAGACACTCGTTGTCCTT
Cx3cr1 mutant	CTCCCCCTGAACCTGAAAC	CCCAGACACTCGTTGTCCTT

Table S3. Primary antibodies

Antibodies	Source	Identifier
Rabbit anti CHIL3	Abcam	Cat# ab192029
Rabbit anti CHI3L1	SAB	Cat# 35680
Rat anti F4/80 (for IF-Frozen)	Abcam	Cat# ab6640
Rabbit anti F4/80 (for IHC-P, IF-P)	CST	Cat# 70076
Rabbit anti CX3CR1	Abcam	Cat# ab8021

Rabbit anti ACTB	CST	Cat# 4970
Rabbit anti CCR1	ABclonal	Cat# A18341
Rabbit anti CCR5	Abcam	Cat# ab7346
Rabbit anti CD206	Abcam	Cat# ab64693
Mouse anti ALAD	Santa Cruz	Cat# sc-271585
Mouse anti ALAS1	Santa Cruz	Cat# sc-137093
Mouse anti p65 NF-κB	Santa Cruz	Cat# sc-8008
Rabbit anti TGF beta 1	ABclonal	Cat# A2124
Rabbit anti Ly-6G	Abcam	Cat# ab25377
Rat anti CD11b	Abcam	Cat# ab128797
PerCP/Cyanine5.5 anti-mouse CD45	Biolegend	Cat# 103131
Brilliant violet 510 anti-mouse/human CD11b	Biolegend	Cat# 101263
APC anti-mouse F4/80	Biolegend	Cat# 123116
Alexa fluor anti-mouse Ly-6C	Biolegend	Cat# 128023
PE anti-mouse CD206	Biolegend	Cat# 141706
Brilliant violet 605 anti-mouse CD86	Biolegend	Cat# 105037

Table S4. Other reagents

Reagents	Source	Identifier
Goat anti-Mouse IgG (H+L) Secondary Antibody, HRP conjugate	Invitrogen	Cat# 31430
Goat anti-Rabbit IgG (H+L) Secondary Antibody, HRP conjugate	Invitrogen	Cat# 31460
Alexa Fluor 488 goat anti-rat IgG (H+L)	Invitrogen	Cat# A-11006
Alexa Fluor 594 goat anti-rat IgG (H+L)	Invitrogen	Cat# A-11007
Alexa Fluor 488 goat anti-mouse IgG (H+L)	Invitrogen	Cat# A-11001
Alexa Fluor 594 goat anti-mouse IgG (H+L)	Invitrogen	Cat# A-11005
Alexa Fluor 488 goat anti-rabbit IgG (H+L)	Invitrogen	Cat# A-11008
BODIPY	Invitrogen	Cat# D3922
MitoNeoD	MedKoo Biosciences	Cat# 563761
Phalloidine 633	Thermo Fisher Scientific	Cat# A22284
TMRE	MCE	Cat# HY-D0985A
Hoechst 33342	Invitrogen	Cat# H3570
DAPI	Invitrogen	Cat# D1306
Tissue-Tek O.C.T Compound	Sakura	Cat# 4583
Taq Pro Universal SYBR qPCR Master Mix	Vazyme	Cat# Q712
HiScript III RT SuperMix for qPCR (+gDNA	Vazyme	Cat# R323

wiper)		
Brewer’s medium	Solarbio	Cat# LA4590
RPMI 1640	Gibco	Cat# C11875500BT
DMEM	Gibco	Cat# C11995500BT
Fetal Bovine Serum	PAN	Cat# P30-3302
M-PER Mammalian Protein Extraction Reagent	Invitrogen	Cat# 78501
T-PER Tissue Protein Extraction Reagent	Invitrogen	Cat# 78510
NuPAGE Sample Reducing Agent (10X)	Invitrogen	Cat# NP0004
NuPAGE LDS Sample Buffer (4X)	Invitrogen	Cat# NP0008
TRIzol	Invitrogen	Cat# 15596026
Mouse Mononuclear Cells Separation Medium	Dakewe	Cat# 7211011
Corn oil	Macklin	Cat# C805618
DMSO	Sigma-Aldrich	Cat# D2650
Collagenase IV	Sigma-Aldrich	Cat# C5138

Table S5. Chemicals, peptides, and recombinant proteins

Chemicals, peptides, and recombinant proteins	Source	Identifier
5-Aminolevulinate	Macklin	Cat# A800543
CHIL3	MCE	Cat# HY-P7845
CHI3L1	MCE	Cat# HY-P70030
IL-4	Peprotech	Cat# 214-14
LPS from *E. coli O111:B4*	Sigma-Aldrich	Cat# L2630
Rotenone	Sigma-Aldrich	Cat# R8875
Decylubiquinone	Sigma-Aldrich	Cat# D7911
NaN_3	Sigma-Aldrich	Cat# S2002
Cytochrome c	Sigma-Aldrich	Cat# C2867
Antimycin A	Sigma-Aldrich	Cat# A8674
BAY-11-7082	MCE	Cat# HY-13453
Betulinic acid	MCE	Cat# HY-10529
T0070907	Selleck	Cat# S2871
Fenofibric acid	Macklin	Cat# 42017-89-0

189 **Table S6. Critical commercial assays**

Critical commercial assays	Source	Identifier
Dako REAL EnVision Detection System, Peroxidase/DAB+ Rabbit/Mouse	Dako	Cat# K5007
TrueView Autofluorescence Quenching kit	Vector	Cat# SP-8500
ECL Chemiluminescent HRP Substrate	Advansta	Cat# K-12045
ECL Ultra Western HRP Substrate	Merck Millipore	Cat# CS222618
Seahorse XF Real-Time ATP Rate Assay Kit	Seahorse	Cat# 103592-100
Mouse YM1/Chitinase 3 like 3 ELISA Kit	RayBio	Cat# ELM-YM1
Mouse IL-1β ELISA Kit	Neobioscience	Cat# EMC001b
Mouse IL-6 ELISA Kit	Neobioscience	Cat# EMC004
Mouse IL-10 ELISA Kit	Neobioscience	Cat# EMC005
Mouse TNF-α ELISA Kit	Neobioscience	Cat# EMC102a
ATP assay kit	Nanjing Jiancheng Bioengineering Institute	Cat# A095-1-1
Lactate assay kit	Solarbio	Cat# BC2230
NADP/NADPH Quantitation Colorimetric Kit	biovison	Cat# K347-100
NAD+/NADH Colorimetric Assay Kit	biovison	Cat# K958-400
Mitochondrial Complex IV activity assay kit	Acmec	Cat# BC0940
Mitochondrial Complex V activity assay kit	Solarbio	Cat# BC1445
CCK-8 Cell Counting Kit	Vazyme	Cat# A311

In Situ Cell Death Detection Kit, Fluorescein	Roche	Cat# 11684795910
NEBNext® UltraTM RNA Library Prep Kit	NEB	Cat# E7775

Table S7. Experimental models

Experimental models	Source	Identifier
Human: L-02	ATCC	CRL-2524
Mouse: B6.129P2(Cg)-Cx3cr1^{tm1Litt}/J	Jackson lab	005582
Mouse: C57BL6/J	Zhuhai BesTest Bio-Tech Co,.Ltd	N/A

Table S8. Software and algorithms

Software and algorithms	Source	Identifier
R v4.0.2	The R Foundation	https://www.r-project.org
Hisat2 v2.0.5	Kim et al. [1]	http://daehwankimlab.github.io/hisat2/
featureCounts package v2.0.1	Liao et al. [2]	https://subread.sourceforge.net/featureCounts.html
DESeq2 package 1.26.0	Love et al. [3]	https://bioconductor.org/packages/release/bioc/html/DES

		eq2.html
Graphpad Prism 8.0	Graphpad	https://www.graphpad.com/
Zen 2.6	Zeiss	https://www.zeiss.com/microscopy/en/products/software/zeiss-zen.html
TissueFAXS SL Viewer 7.0.6245	Tissuegnostics	https://tissuegnostics.com/products/scanning-and-viewing-software/tissuefaxs-viewer
Oxymax/CLAMS	Columbus	https://www.colinst.com/downloads/?p=o
ImageJ 1.53	National Institutes of Health	https://imagej.nih.gov/ij/
CytExpert 2.4	Beckman	https://www.beckman.com/flow-cytometry/research-flow-cytometers/cytoflex/software

Supplemental References

1. Kim D, Paggi JM, Park C, Bennett C, Salzberg SL. Graph-based genome alignment and genotyping with HISAT2 and HISAT-genotype. Nature Biotechnology. 2019; 37: 907-15.
2. Liao Y, Smyth GK, Shi W. featureCounts: an efficient general purpose program for assigning sequence reads to genomic features. Bioinformatics. 2013; 30: 923-30.
3. Love MI, Huber W, Anders S. Moderated estimation of fold change and dispersion for RNA-seq data with DESeq2. Genome Biology. 2014; 15.
